# Bioaccessibility via In Vitro Digestion and Colonic Fermentation, Intake, and per Serving Risk Assessment of Mn, Co, and Ni in Cooked Food

**DOI:** 10.3390/foods15203599

**Published:** 2026-10-10

**Authors:** Miguel Navarro-Moreno, José Ángel Rufián-Henares, Konstantinos Douros, Francisco M. Ocaña-Peinado, Miguel Navarro-Alarcón, Silvia Pastoriza

**Affiliations:** 1 Department of Nutrition and Food Science, School of Pharmacy, University of Granada, 18071 Granada, Spain; miguelnav@ugr.es (M.N.-M.); jarufian@ugr.es (J.Á.R.-H.); spdelacueva@ugr.es (S.P.); 2 Institute of Nutrition and Food Technology, INyTA (IBS), University of Granada, 18071 Granada, Spain; 3 PhD Program in Nutrition and Food Sciences, University of Granada, 18071 Granada, Spain; 4 Pediatric Allergy and Respiratory Unit, 3rd Department of Pediatrics, School of Medicine, “Attikon” University Hospital, National and Kapodistrian University of Athens, 11527 Athens, Greece; kostas.douros@gmail.com; 5 Department of Statistics and Operations Research, School of Pharmacy, University of Granada, 18071 Granada, Spain; fmocan@ugr.es

**Keywords:** home culinary techniques, in vitro digestibility, INFOGEST, childhood pathologies, gluten-related disorders, obesity, allergy/intolerance to cow’s milk protein, health hazard

## Abstract

Manganese (Mn), cobalt (Co), and nickel (Ni) are trace elements whose nutritional and toxicological effects depend on their bioaccessibility (BA). This study evaluated the effects of food matrix, cooking, life stage, and childhood health status on Mn, Co, and Ni BA and estimated dietary exposure per serving. A total of 55 commonly consumed raw and cooked plant- and animal-based foods were analyzed. BA was assessed using a standardized in vitro oral–gastrointestinal digestion model followed by colonic fermentation with fecal inoculum from healthy adults and children. Metal concentrations were determined by ICP-MS/MS, and exposure was compared with nutritional and toxicological reference values. Plant-based foods showed significantly higher total MnBA and NiBA than animal-based foods (34.8 vs. 19.8% and 51.4 vs. 42.3%, respectively), whereas cooking generally increased MnBA, CoBA, and NiBA (expressed as the solubilized fraction available for absorption). Colonic fermentation substantially contributed to trace element BA, highlighting the role of gut microbiota. Healthy children exhibited higher colonic MnBA and CoBA than adults, while celiac and allergic children showed greater MnBA than healthy children. Mn and Co intakes remained below toxicological concern, whereas several foods provided relatively high Ni intakes, particularly for children. Overall, food composition, culinary processing, and gut microbiota influenced trace element BA and dietary risk assessment.

## 1. Introduction

Foods are important dietary sources of essential trace elements such as Mn [1,2] and, to a lesser extent, Co, which is required only in trace amounts. Average dietary Co intake in the United Kingdom has been estimated at 12 µg/day [3], whereas a beneficial daily intake of 25–35 µg has been suggested for Ni [4].

Mn acts as a cofactor for numerous enzymes, including pyruvate carboxylase, mitochondrial superoxide dismutase, and arginase, which are involved in bone development, growth, and the metabolism of carbohydrates, lipids, and proteins [5,6]. However, excessive Mn exposure, particularly among strict vegetarians consuming Mn supplements, has been associated with neurotoxicity, gut microbiota dysbiosis, intestinal inflammation [7], and Parkinsonian-like neurological alterations. Infants with cow’s milk protein allergy fed soy-based formulas also warrant particular attention because of the high Mn content and enhanced absorption of these products [8].

Cobalt plays an essential role in the intestinal microbial synthesis of cobalamin (vitamin B_12_) [9]. The U.S. EPA has established a provisional chronic oral reference dose (RfD) of 0.0003 mg/kg body weight/day [10]. In experimental animals, excessive Co exposure has been associated with reduced feed intake, anemia, and hepatic accumulation [9]. Cobalt can cause multisystem toxicity [11]. Acute exposure is relatively uncommon and is most often associated with excessive supplementation or accidental ingestion, whereas chronic exposure is more commonly related to occupational settings [11]. The main routes of human exposure to Co include occupational and environmental exposure, as well as oral exposure through the diet, dietary supplements, and certain medications. Excessive Co intake has been associated with several adverse health effects, including neurological disorders, cardiomyopathy, and endocrine disturbances, particularly alterations in thyroid function and hypothyroidism [11,12]. The relationship between Co status and obesity remains inconclusive, with studies reporting both protective [13] and adverse [14] associations.

Regarding Co and Mn, studies in edible plants have shown that exposure assessments based on bioaccessibility (BA) provide lower estimates than those based on total elemental concentrations. Consequently, the estimated dietary exposure and the associated potential health risk are reduced, as previously reported for Co [15].

Nickel, although generally regarded as a non-essential trace element [16], has been associated with allergic contact dermatitis [17,18] and carcinogenic effects following chronic exposure [5]. Elevated exposure to Ni compounds has been linked to the induction of oxidative stress, impairment of DNA repair mechanisms, and subsequent carcinogenesis [19].

In Europe, chronic dietary Ni exposure has been estimated to range from 3.05–14.6 µg/kg body weight/day in children and 1.83–4.19 µg/kg body weight/day in adults [20]. Foods with relatively high Ni concentrations are predominantly plant-based, particularly chocolate, spices, nuts, and legumes, as well as confectionery, sweeteners, and stimulant foods [5]. Excessive exposure to Ni has been associated with impaired growth and neurodevelopment [21] and an increased cancer risk associated with rice consumption [22]. In patients with celiac disease, long-term adherence to a gluten-free diet may also exacerbate Ni-induced symptoms [23].

Conversely, several studies evaluating dietary exposure to Mn, Co, and Ni from a wide range of food products have concluded that their consumption does not pose a significant health risk under current dietary exposure levels [24,25,26]. Before consumption, foods undergo household cooking processes that influence their composition and nutritional value [26,27,28,29,30,31,32,33,34,35,36,37,38,39,40]. These processes alter the original structure, texture, and chemical composition of foods through protein denaturation, lipid oxidation, Maillard reactions, and the degradation or formation of new compounds that may act as ligands and influence the intestinal solubility of minerals, thereby affecting their BA. In vitro studies have reported reductions of up to 27% in the bioaccessibility of Mn (MnBA) in roasted chickpeas, with greater decreases occurring at longer roasting times and higher temperatures [41]. Higher concentrations of Mn and Ni have been reported in raw beef cuts than in cooked samples [42]. The release of Ni from cookware decreases with repeated use and is greater from stainless steel [43,44]. However, cooking rice in stainless steel cookware does not appear to affect Ni bioaccessibility (NiBA), nor is it influenced by the protein or fiber content of the food [45]. Parboiling has been shown to reduce Mn concentrations in rice [46]. During pea preparation, approximately 25% of Co may be lost through leaching, whereas Mn losses are minimal [47]. Similarly, reductions in Mn and Ni concentrations have been reported during the preparation of bean cakes cooked in aluminum foil [48]. Another study investigated the effects of different cooking methods on the mineral composition and BA of liver from various animal species, including Mn and Co, and concluded that *sous-vide* cooking was the most effective method for preserving its nutritional quality [31]. Importantly, the bioaccessible fraction of trace elements, rather than their total concentration, is more relevant for assessing their nutritional value and potential toxicological effects [15,28].

In vivo studies are impractical for evaluating multiple foods, household cooking techniques, and trace elements such as Co, which occurs in different chemical species [49]. In contrast, in vitro methods simulate oral, gastric, and small-intestinal digestion using the harmonized INFOGEST 2.0 protocol [50] and, when combined with a colonic fermentation stage using human fecal inoculum [28,29,30], enable the estimation of the bioaccessible fraction available for intestinal absorption [45]. More advanced systems, such as the Simulator of the Human Intestinal Microbial Ecosystem (SHIME), provide a more comprehensive simulation of human gastrointestinal physiology, although they require prolonged microbiota stabilization and extensive experimental preparation.

The composition of the gut microbiota is influenced by environmental factors, particularly diet, and the intestine is a highly plastic organ that rapidly adapts to dietary changes and exposure to trace elements [6,51]. Consequently, both life stage and childhood disorders may modulate gut microbiota composition and function, thereby influencing the bioaccessibility of Mn, Co, and Ni (MnBA, CoBA, and NiBA, respectively; expressed as the solubilized fraction available for absorption). Accordingly, we hypothesized that the BA of these trace elements is affected by household cooking techniques, life stage (adults vs. children), and childhood disorders, including gluten-related disorders, obesity, and allergy/intolerance to cow’s milk proteins. To test this hypothesis, an in vitro oral-gastrointestinal digestion model coupled with colonic fermentation using human fecal inoculum was applied to a wide range of raw and cooked foods prepared by frying, roasting, toasting, boiling, and grilling. The bioaccessible and non-bioaccessible fractions of Mn, Co, and Ni were determined in the small and large intestine, and the estimated intakes per serving were compared with established reference values to assess their potential health risks.

## 2. Materials and Methods

### 2.1. Food Samples and Cooking Techniques

A total of 159 samples from 55 foods (122 plant-based, 37 animal-based [28] were analyzed; Table S1). Six culinary techniques were used: raw, roasting, frying, toasting (only for whole-grain and white bread), boiling, and grilling [52]. Foods were analyzed in the form in which they are typically consumed by the population. The ratios and times for food cooking were adapted accordingly [53]. For frying, food was heated at 180 °C for 8 min using an extra virgin olive oil/food ratio of 5:1. For grilling, temperatures between 220–250 °C were applied for 3 min with an oil/food ratio of 0.5:1. For boiling, food was heated at 100 °C for 20 min with a water/food ratio of 5:1. For roasting, food was heated at 180 °C for 10 min. Toasting was conducted using the fourth setting (900 W for 3 min) on a Grunkel TS140H toaster (Sevilla, Spain). Details on sample-to-treatment ratios and utensils used are available elsewhere [52,53]. All samples were homogenized and stored at −80 °C in a nitrogen atmosphere prior to analysis.

### 2.2. Stool Samples

To study bioaccessibility in the large intestine (BA-LI), stool samples were obtained from previously characterized groups: healthy adults (HE-AD; *n* = 10), healthy children (HE-CH; *n* = 10), and children with gluten-related disorders (GRD-CH; *n* = 10), obesity (OB-CH; *n* = 10), and allergy/intolerance to cow’s milk protein (AICM-CH; *n* = 10) [27,54]. The specific eligibility criteria for the five study populations included in the study are detailed elsewhere [54]. The five study populations were as follows:

(i) Apparently healthy adult subjects (both sexes), aged 20–65 years, with a body mass index (BMI) between 20–28 kg/m^2^, stable weight, ability to use a smartphone, and Internet connection availability. Exclusion criteria are the diagnosis of chronic gastrointestinal (GI) disorders, coeliac disease, or chronic diseases such as diabetes or other metabolic diseases; present pregnancy or lactation (<6 weeks before study start), or intention to become pregnant in the next 12 weeks; recent inflammation and/or long-term use of anti-inflammatory drugs; medically prescribed diet or specific dietary regimens for any reasons (i.e., high-protein diet, vegetarianism, veganism, etc.); antibiotic treatment (<3 months before study start); intake of antioxidant, pre- or probiotic supplements (<1 month before study start); intense physical activity (>10 h/week); alcohol consumption >21 drinks/week for men and >14 drinks/week for women.

(ii) Normal weight children (of both sexes), aged 6–11 years, with a BMI >5th and <85th percentile for age, gender, and height. Exclusion criteria are the diagnosis of chronic GI disorders and any other chronic disease, elimination diet, and intake of probiotics (<2 weeks before study start).

(iii) Children (of both sexes) with obesity, aged 6–11 years, with a BMI ≥95th percentile for age, gender, and height. Exclusion criteria are the diagnosis of chronic GI disorders and endocrinopathies, and intake of probiotics (<2 weeks before study start).

(iv) Children (of both sexes) with gluten-related disorders, aged 6–11 years, diagnosed with coeliac disease or wheat allergy (sIgE), on an elimination diet. The exclusion criteria are the absence of symptoms.

(v) Children (of both sexes) with allergy/intolerance to cow’s milk, aged 6–11 years, with an IgE-mediated milk allergy on an elimination diet from infancy, lactose intolerance (symptoms plus breath hydrogen positive test), overgrown IgE-mediated milk allergy but current aversion to milk, or non-definable phenotype (children who avoid milk but do not belong to any of the previous phenotypes). Exclusion criteria are the diagnosis of other GI comorbidities.

Prior to collection, written informed consent was obtained from adults or the legal guardians of children. Sufficient fecal material was collected for in vitro fermentation. Fecal samples from individual donors were analyzed separately. The consent form detailed the study procedures, inclusion/exclusion criteria, and potential risks. The study was conducted according to the Declaration of Helsinki and approved by the Ethics Committee of the University of Granada (protocol 1080/CEIH/2020; date of approval: 10 June 2020) and, in Greece, by the Scientific Committees of the University Hospital of Ioannina (protocol 382, decision 10/3 June 2020), University Hospital “Attikon” (decision 546/1 October 2020), and University Hospital of Patras (decision 360/22 July 20).

### 2.3. In Vitro Procedure for Bioaccessibility Assessment

Food samples were subjected to a previously optimized in vitro procedure that simulates physiological digestion in three phases: oral, gastric, and intestinal [55,56]; Table S2). At the end of the intestinal phase, tubes were kept on ice to stop enzymatic reactions and centrifuged at 3500 rpm for 10 min. The supernatant, representing the solubilized fraction available for absorption in the small intestine (MnBA-SI, CoBA-SI, and NiBA-SI), was stored in 1 mL tubes at −80 °C until analysis.

Subsequently, in vitro fermentation was carried out using fecal inoculum from donors (HE-AD, HE-CH, GRD-CH, OB-CH, and AICM-CH). The solid pellet obtained after oral-gastrointestinal digestion, combined with 10% of the digestion supernatant, was fermented (Table S2) [28,29,56]. At the end of fermentation, tubes were kept on ice to stop microbial activity and centrifuged at 3500 rpm for 10 min. The supernatant obtained represented the solubilized fraction available for absorption in the large intestine (MnBA-LI, CoBA-LI, and NiBA-LI), while the pellet corresponded to the insolubilized non-bioaccessible fraction excreted in the feces.

All fractions were stored at −80 °C until analysis. The in vitro procedure was performed in triplicate for each food sample. In summary, three fractions were obtained: (i) MnBA-SI, CoBA-SI, and NiBA-SI, corresponding to the solubilized bioaccessible fractions recovered from the digestion supernatant; (ii) MnBA-LI, CoBA-LI, and NiBA-LI, corresponding to the solubilized bioaccessible fractions recovered from the fermentation supernatant; and (iii) insoluble non-bioaccessible Mn, Co, and Ni, corresponding to the fractions recovered from the fermentation pellet. Accordingly, the total Mn, Co, and Ni content of each food sample was calculated as the sum of the respective small-intestinal, large-intestinal, and non-bioaccessible fractions.

Results are expressed as mg Mn/kg, µg Co/kg, and µg Ni/kg of food (fresh weight, f.w.).

### 2.4. Mineralization of Obtained Fractions and Trace Metal Analysis

For Mn, Co and Ni determination, a microwave-assisted acid digestion protocol was used [27]. For digestion supernatants, 250 mg of sample was treated with 3.5 mL of 33% HNO_3_ in 25 mL Falcon tubes and left for 96 h. Samples were diluted to 25 mL with Milli-Q water. For fermentation pellets and certain supernatants (50–100 mg), 3 mL of 65% HNO_3_ was added in borosilicate tubes. Additionally, 0.5 mL of 65% HNO_3_ and 2.5 mL of Milli-Q water were placed into microwave vessels, into which the borosilicate tubes were placed. Mineralization was performed in Teflon vessels using a Multiwave 5000 microwave digester with Rotor 24HVT50 (Anton Paar GmbH, Graz, Austria) under previously optimized conditions [27,57]. Final solutions were diluted to 40 mL with Milli-Q water. Four blanks per batch were included.

Trace metal concentrations in the resulting solutions were measured using inductively coupled plasma mass spectrometry (ICP-MS/MS; Agilent 8900, Agilent Technologies Inc., Santa Clara, CA, USA). Calibration curves for Mn, Co, and Ni were prepared from standard solutions of 1000 mg/L in 1% HNO_3_ (Merck, Darmstadt, Germany). Quantification was performed by external calibration using three analyte-specific calibration curves. The linear calibration ranges were 0.250–250 µg/L for Mn (*y* = 0.0650*x* + 0.0011; *R*^2^ = 0.9999), 0.025–25.0 µg/L for Co (*y* = 0.3113*x* + 0.0003; *R*^2^ = 0.9999), and 0.050–50.0 µg/L for Ni (*y* = 0.0833*x* + 0.0070; *R*^2^ = 0.9999). In the calibration equations, *y* denotes the instrument response (counts per second, CPS) and *x* the analyte concentration (µg/L). Internal standards (Ge, Ir, Rh, Sc; ISC Science, batch 20210712) were used during measurements. All analyses were performed in triplicate using a linear calibration method.

### 2.5. Checking the Analytical Parameters

Analytical performance characteristics were evaluated prior to sample analysis. The limits of detection (LOD) and limits of quantification (LOQ) for Mn, Co, and Ni were calculated as three and ten times, respectively, the standard deviation of ten reagent blanks divided by the slope of the corresponding calibration curve (Table S3). The method’s accuracy was assessed using three certified reference materials (CRMs): NIST 1515 Apple Leaves (National Institute of Standards and Technology, Gaithersburg, MD, USA), ERM-CE278k Mussel Tissue (European Reference Materials, Geel, Belgium), and BCR-679 White Cabbage (Community Bureau of Reference, Geel, Belgium).

Potential matrix effects were further evaluated by the standard addition method using three representative food matrices. After determination of the native analyte concentrations, samples were fortified with single-element standard solutions to approximately twice their original concentrations. Method selectivity was verified by statistically comparing the slopes of the external calibration curves with those obtained by the standard addition method for each analyte (Table S3).

Method precision was assessed as repeatability (intra-day precision) by analyzing seven independent replicates (*n* = 7) of each of the three representative food matrices. Precision was expressed as the relative standard deviation (%RSD) (Table S3).

### 2.6. Quality Assurance of the Analytical Method

Excellent linearity was obtained for all calibration curves, with coefficients of correlation (*R*^2^) equal to 0.9999. The LOD ranged from 0.001 µg/L for Co to 0.032 µg/L for Mn, whereas LOQ ranged from 0.003 µg/L for Co to 0.096 µg/L for Mn. The corresponding LOD and LOQ values for Ni were 0.016 and 0.048 µg/L, respectively (Table S3).

The method’s accuracy was confirmed by the analysis of CRMs, which yielded recoveries ranging from 95.6 to 106.7% (Table S3). In addition, recovery values obtained from spiked samples subjected to the complete mineralization and analytical procedure ranged from 90.3 to 110.1%, indicating negligible matrix effects. Method precision, expressed as RSD, ranged from 4.23 to 12.1% for Mn, Co, and Ni. Overall, these results demonstrate the accuracy, precision, and robustness of the proposed analytical method for the determination of trace levels of Mn, Co, and Ni in the analyzed food matrices.

### 2.7. Assessment of Metal Intake and Risk per Serving

The metal intake per serving (MIS, for Mn, Co, and Ni) for the analyzed foods was calculated using the following formula:

MIS = (MC × SW)/10^3^

where: MC is the metal concentration (Mn expressed in mg/kg; Co and Ni expressed in µg/kg); SW is the serving weight (g), according to SENC data [58].

For Mn, the percentage coverage (%) of the dietary reference intakes (DRIs; 1.5, 1.8, and 2.3 mg/day for children aged 3–8 years, adult females, and adult males, respectively; [59]) was calculated for the intake per serving of each food using the following formula:

%DRIs = (MISMn × 100%)/DRIsMn

For Co, since no DRIs are established, the provisional percentage coverage of the RfD (considered safe) was calculated. The RfD is 0.0003 mg/kg-day [10], corresponding to 21 µg/day for healthy adults (70 kg) and 5.4–8.1 µg/day for children aged 4–8 years (18–27 kg). The percentage coverage of the RfD for each food serving was calculated using the measured Co content and the serving size.

%RfD = (MISCo × 100%)/RfdCo

For Ni, only the tolerable upper intake levels (TUIL) for humans are established: 300 µg/day for children aged 4–8 years and 1000 µg/day for healthy adults [4]. The percentage coverage of the TUIL for each food serving was calculated using the measured Ni concentration and the corresponding serving size.

%TUIL = (MISNi × 100%)/TUILNi

### 2.8. Statistical Analysis

Statistical analyses were performed using IBM SPSS Statistics (version 28.0; IBM Corp., Armonk, NY, USA). Data are presented as mean ± standard deviation (SD) for Mn, Co, and Ni concentrations, BA values (expressed as solubilized fraction available for absorption), and dietary risk assessment indices. Prior to statistical analysis, data normality and homogeneity of variances were assessed using the Kolmogorov–Smirnov and Levene’s tests, respectively. As the assumptions for parametric analyses were not consistently fulfilled (*p* < 0.05), non-parametric statistical methods were applied throughout the study. Regarding variability, all individual data obtained for each food sample and each subject included in the study were considered in the analysis.

The Kruskal–Wallis test was used to evaluate overall differences among multiple independent groups (e.g., food categories, cooking techniques, digestion phases or population groups). When significant differences were detected, pairwise comparisons were performed using the Mann–Whitney U test. Statistical significance was established at *p* < 0.05. Comparisons between the BA values obtained for the small intestine and the large intestine were performed using statistical analyses appropriate for paired data, specifically the Wilcoxon signed-rank test. Similarly, comparisons between raw and thermally processed samples from the same food batches were also performed using the Wilcoxon signed-rank test.

Principal component analysis (PCA) was performed to explore multivariate relationships among trace metal bioaccessibility values across different food matrices and donor groups. The analysis was conducted on raw individual sample data, without averaging observations by donors or food groups. PCA was performed using the correlation matrix to investigate relationships among trace metal bioaccessibility values across the different food matrices and donor groups. Data were analyzed using the Pearson correlation matrix, which inherently standardizes the variables (Z-score auto-scaling to zero mean and unit variance) prior to component extraction, thereby eliminating scale dependencies arising from differing elemental concentration magnitudes. Principal components were retained according to Kaiser’s criterion (eigenvalues > 1.0) and the cumulative percentage of explained variance. To improve the interpretability of the extracted components, Varimax orthogonal rotation was applied. Component scores and variable loadings were used to examine sample clustering and variable contributions, and the results were visualized using two- and three-dimensional scores and loading plots.

Figures were prepared using Microsoft PowerPoint 2010 (Microsoft Corporation, Redmond, WA, USA).

## 3. Results and Discussion

### 3.1. Bioaccessibility of Mn, Co, and Ni in Food

**Figure 1:**
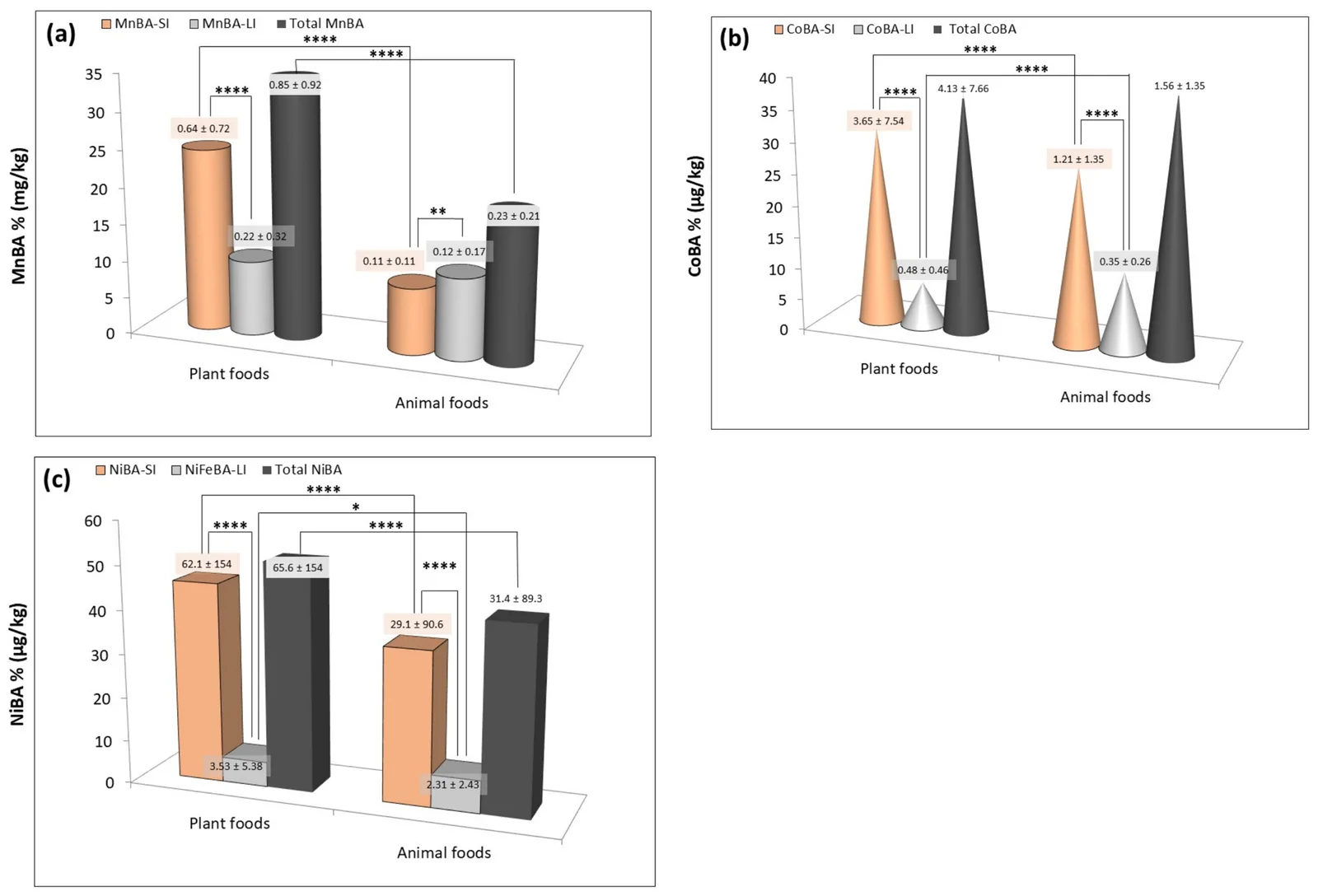
Mean total and partial bioaccessibility values (expressed as solubilized fraction available for absorption, %; total BA; BA in the small intestine and in the large intestine: BA-SI and BA-LI, respectively) of the trace metals analyzed in plant- and animal-based foods: (**a**) Mn bioaccessibility (MnBA); (**b**) Co bioaccessibility (CoBA); (**c**) Ni bioaccessibility (NiBA). Asterisks indicate statistically significant differences (* *p* < 0.05, ** *p* < 0.01, **** *p* < 0.0001).

The BA of Mn, Co, and Ni varied considerably among the analyzed foods (Figure 1a–c; the corresponding box-and-whisker plots are provided in the Supplementary Material as Figure S1a–f), reflecting differences in food origin, matrix composition, and the culinary techniques applied prior to consumption [28,29,30,41,60,61].

In plant-based foods, the small-intestinal bioaccessibility (BA-SI) of Mn, Co, and Ni (24.7 ± 17.2%, 31.2 ± 21.3%, and 45.8 ± 27.0%, respectively; expressed as solubilized fraction available for absorption) was significantly higher than their corresponding large-intestinal bioaccessibility (BA-LI) values (10.1 ± 13.3%, 6.89 ± 6.77%, and 5.68 ± 7.50%, *p* < 0.0001; Figure 1a–c; respectively). Our results agree with those reported by Cai et al. [60], who found that MnBA decreased during the colonic fermentation phase compared with the gastric and small-intestinal phases in ten vegetable matrices. These findings indicate that, in plant-derived foods, the majority of Mn, Co, and Ni becomes bioaccessible during the upper gastrointestinal phase. Nevertheless, the contribution of the colonic phase remains substantial, particularly for Mn, suggesting that microbial fermentation and the formation of soluble metal complexes contribute to the overall BA of these trace elements.

In animal-based foods, Co and Ni also exhibited significantly lower bioaccessibility in the large intestine than in the small intestine (CoBA-LI: 12.1 ± 12.4% vs. CoBA-SI: 27.5 ± 21.1%; NiBA-LI: 7.43 ± 10.3% vs. NiBA-SI: 34.9 ± 25.0%; Figure 1b,c; *p* < 0.0001; the corresponding box-and-whisker plots are provided in the Supplementary Material as Figure S1c,e; respectively). In contrast, Mn showed a modest but significantly higher BA during the colonic phase than during the small-intestinal phase (MnBA-LI: 10.9 ± 15.8% vs. MnBA-SI: 8.88 ± 9.77%; Figure 1a; *p* < 0.0001; the corresponding box-and-whisker plots are provided in the Supplementary Material as Figure S1a). Although most trace metal BA was established during the small-intestinal phase, the colonic contribution was substantial, particularly for Mn in animal-derived foods (Figure 1a). These findings suggest that microbial fermentation may enhance the solubilization of certain trace metals through the production of short-chain fatty acids (SCFAs) and other low-molecular-weight metabolites capable of forming soluble metal complexes [60,62].

The higher total bioaccessibility of Mn and Ni observed in plant-based foods (Figure 1a,c) may be attributed to differences in food matrix composition. Components such as proteins, dietary fiber, polyphenols, ascorbic acid, and phytic acid can either promote or inhibit mineral release during digestion, depending on their concentration and chemical interactions within the food matrix [63]. Further studies are required to elucidate the relative contribution of these factors.

Within plant-based foods, protein-rich matrices, including legumes, nuts, and cereals, exhibited significantly higher total MnBA but lower total NiBA than the remaining food groups (Table S4; *p* < 0.0001). Previous studies support these observations, showing that soy protein increased total Mn bioaccessibility in cruciferous vegetables, whereas casein and lactalbumin had little effect on Mn but significantly reduced NiBA, probably through the formation of poorly soluble protein–metal complexes [64]. Throughout the manuscript, it should be noted that the concentration reported for each bioaccessible fraction after digestion and fermentation is the measured concentration of the element in the soluble fraction, whereas bioaccessibility is expressed as the percentage of the total amount of the element that remained soluble following the in vitro procedure applied in the study.

As shown in Table 1, significant differences (*p* < 0.05) were also observed among the different categories of plant-based foods for both fractional and total bioaccessibility of Mn, Co, and Ni. The ‘“other foods” and nuts’ categories had the highest total MnBA, CoBA, and NiBA values (*p* < 0.0001). During the colonic phase, the highest MnBA-LI values were found in nuts (15.6 ± 15.5%; 0.57 ± 0.63 mg/kg) and legumes (13.1 ± 15.4%; 0.23 ± 0.26 mg/kg) (*p* < 0.0001). In contrast, CoBA-LI was greatest in fruits (9.89 ± 7.74%; 0.47 ± 0.36 μg/kg) and vegetables (7.83 ± 6.44%; 0.53 ± 0.42 μg/kg) (*p* < 0.0001), whereas the highest NiBA-LI values were observed in vegetables (7.36 ± 8.36%; 4.25 ± 6.68 μg/kg) and fruits (7.37 ± 7.77%; 3.13 ± 3.66 μg/kg) (*p* < 0.0001). These findings indicate that the food matrix is a major determinant of trace metal BA and that different food categories differentially modulate the release of Mn, Co, and Ni throughout gastrointestinal digestion and colonic fermentation.

Regarding dietary fiber, whole-grain products exhibited significantly higher total CoBA than refined cereals (Table S5). Moreover, the large-intestinal bioaccessibility of all three trace metals was significantly greater in whole grains than in refined cereals (MnBA-LI: 9.58 ± 12.5% vs. 6.12 ± 8.20%; CoBA-LI: 4.61 ± 6.13% vs. 2.34 ± 2.50%; NiBA-LI: 2.73 ± 4.29% vs. 1.96 ± 3.52%; *p* < 0.01). In addition, CoBA-SI was also significantly higher in whole-grain products (23.3 ± 22.7% vs. 17.4 ± 15.3%; *p* = 0.021).

The influence of dietary fiber on trace metal bioaccessibility remains controversial. Marcondes Luz et al. [61] reported that neither dietary fiber nor myo-inositol phosphates significantly affected Mn bioaccessibility in plant-based burgers. In contrast, Ortiz and Cámara-Martos [65] found negative associations between fiber and protein contents and MnBA in rice. Likewise, Silvério Filbido et al. [63] demonstrated that phytic acid and dietary fiber reduced MnBA in fortified infant foods, whereas ascorbic acid exerted a positive effect. For Ni, however, Ortiz et al. [45] observed no significant influence of either dietary fiber or protein content on its bioaccessibility in different rice varieties. These apparently contradictory findings suggest that the effect of dietary fiber depends not only on its quantity but also on its physicochemical characteristics, its interactions with other food components, and the complexity of the food matrix.

Similarly, gluten-containing cereals exhibited significantly higher BA-LI values for Mn, Co, and Ni than gluten-free cereals (Table S6; *p* = 0.001). In addition, MnBA-SI was also significantly higher in gluten-containing cereals (*p* < 0.0001). This pattern is consistent with enhanced colonic fermentation of soluble dietary fiber and gluten-derived substrates, leading to increased production of SCFAs and a reduction in luminal pH [55]. These conditions may increase the solubility of Mn, Co, and Ni through the formation of soluble metal complexes, thereby enhancing their BA during the colonic phase. Supporting this hypothesis, others [64] reported that soluble fibers such as pectin and gum arabic increased MnBA, whereas no comparable effect was observed for Ni.

The highest Ni concentrations were found in plant-based foods (Table 1), in agreement with previous studies reporting greater Ni accumulation in plant matrices than in foods of animal origin [66]. Since these foods also exhibited the highest NiBA (Figure 1c), they may represent the principal dietary contributors to bioaccessible Ni intake and, consequently, to potential health risks associated with excessive exposure.

It should be emphasized that most published studies on trace metal BA have evaluated a limited number of samples and have generally focused on a single food group or a restricted range of food matrices, such as vegetables [60], rice [65], fortified infant foods [63], plant-based yoghurts [67], or plant-based burgers [61]. In contrast, the present work provides a comprehensive assessment across a wide variety of plant- and animal-derived foods, allowing broader conclusions regarding the influence of food matrix and gastrointestinal digestion on Mn, Co, and Ni bioaccessibility (expressed as solubilized fraction available for absorption).

As shown in Table 1, significant differences (*p* < 0.05) were observed among the different categories of animal-based foods. Dairy products and lamb exhibited the highest total MnBA (*p* < 0.0001), whereas pork and chicken showed the highest total CoBA (*p* < 0.0001). Total NiBA was greatest in pork and salmon (*p* < 0.0001). During the colonic phase, the highest MnBA-LI values were found in lamb (20.5 ± 21.4%; 0.17 ± 0.17 mg/kg) and dairy products (14.5 ± 19.7%; 0.13 ± 0.16 mg/kg) (*p* < 0.0001). Similarly, the highest CoBA-LI values were observed in lamb (27.0 ± 15.8%; 0.55 ± 0.26 μg/kg) and pork (15.6 ± 14.7%; 0.39 ± 0.24 μg/kg) (*p* < 0.0001), whereas NiBA-LI was greatest in lamb (10.9 ± 12.3%; 2.70 ± 2.27 μg/kg) and dairy products (9.02 ± 14.0%; 2.51 ± 2.74 μg/kg) (*p* < 0.0001).

Dairy products were particularly noteworthy because they consistently exhibited high MnBA during both the small- and large-intestinal phases, resulting in the highest total MnBA values among the animal-derived foods. This observation agrees with previous studies reporting that enzymatic protein hydrolysis in infant formulas and whole milk had little influence on MnBA and that replacing whey proteins with soy proteins did not significantly modify Mn dialysability [68]. The consistently high MnBA observed in dairy products may therefore be more closely related to the intrinsic characteristics of the dairy matrix than to protein composition alone. In addition, milk proteins, particularly casein-derived phosphor-peptides generated during digestion, together with lactose, are known to maintain divalent cations in soluble forms, thereby favoring mineral BA and potentially contributing to the elevated MnBA values observed in the present study.

Table S7 summarizes the fractional and total BA values of Mn, Co, and Ni in vegetables (*n* = 59) and fruits (*n* = 25), which together accounted for more than half of the food samples analyzed. Among vegetables, spinach and garlic exhibited the highest total MnBA (57.6 ± 18.4% and 52.0 ± 23.6%, respectively). Combined with their relatively high Mn concentrations, these foods represent important dietary sources of bioaccessible Mn, in agreement with previous findings for spinach [60].

Table S8 summarizes the concentrations and bioaccessibility values of Mn, Co, and Ni reported in foods that are also included in the present study, together with the corresponding daily dietary intakes and risk assessments described in previous studies. Table S8 illustrates the wide variability in the concentrations and BA of Mn, Co, and Ni reported across different studies and countries [24,25,31,41,60,61,63,65,66,67,69,70,71,72,73] in agreement with the findings of the present study.

For Co and Ni, the highest total BA values were observed in cabbage and bell peppers (64.2 ± 17.2% and 60.7 ± 21.8% for Co; 67.4 ± 12.6% and 67.5 ± 15.6% for Ni, respectively). Among fruits, grapes and bananas showed the highest total MnBA and CoBA, whereas plums and peaches exhibited the highest total NiBA (Table S7).

Overall, the BA of Mn, Co, and Ni varied considerably among foods, reflecting differences in food origin and matrix composition. In plant-based foods, BA was established predominantly during the small-intestinal phase for all three trace metals. A similar pattern was observed for Co and Ni in animal-based foods. Nevertheless, the contribution of the large-intestinal phase was also substantial, particularly for Co and Ni in plant-based foods and for Mn in animal-derived foods. These findings suggest that colonic fermentation may contribute to the release or solubilization of these trace metals, potentially through the production of SCFAs and other microbial metabolites capable of forming soluble metal complexes.

At the individual food level, whole-grain and gluten-containing cereal products exhibited significantly higher BA-LI values for all three trace metals, consistent with their greater fermentable substrate content and the metabolic activity of the colonic microbiota. Furthermore, the combination of relatively high Mn concentrations and elevated total MnBA in spinach and garlic identifies these foods as important contributors to dietary Mn intake. Although these foods represent valuable sources of this essential trace element, excessive consumption—particularly in susceptible population groups—could increase Mn exposure and therefore warrants consideration in dietary risk assessments.

### 3.2. Bioaccessibility of Mn, Co, and Ni According to Household Cooking Techniques

**Figure 2:**
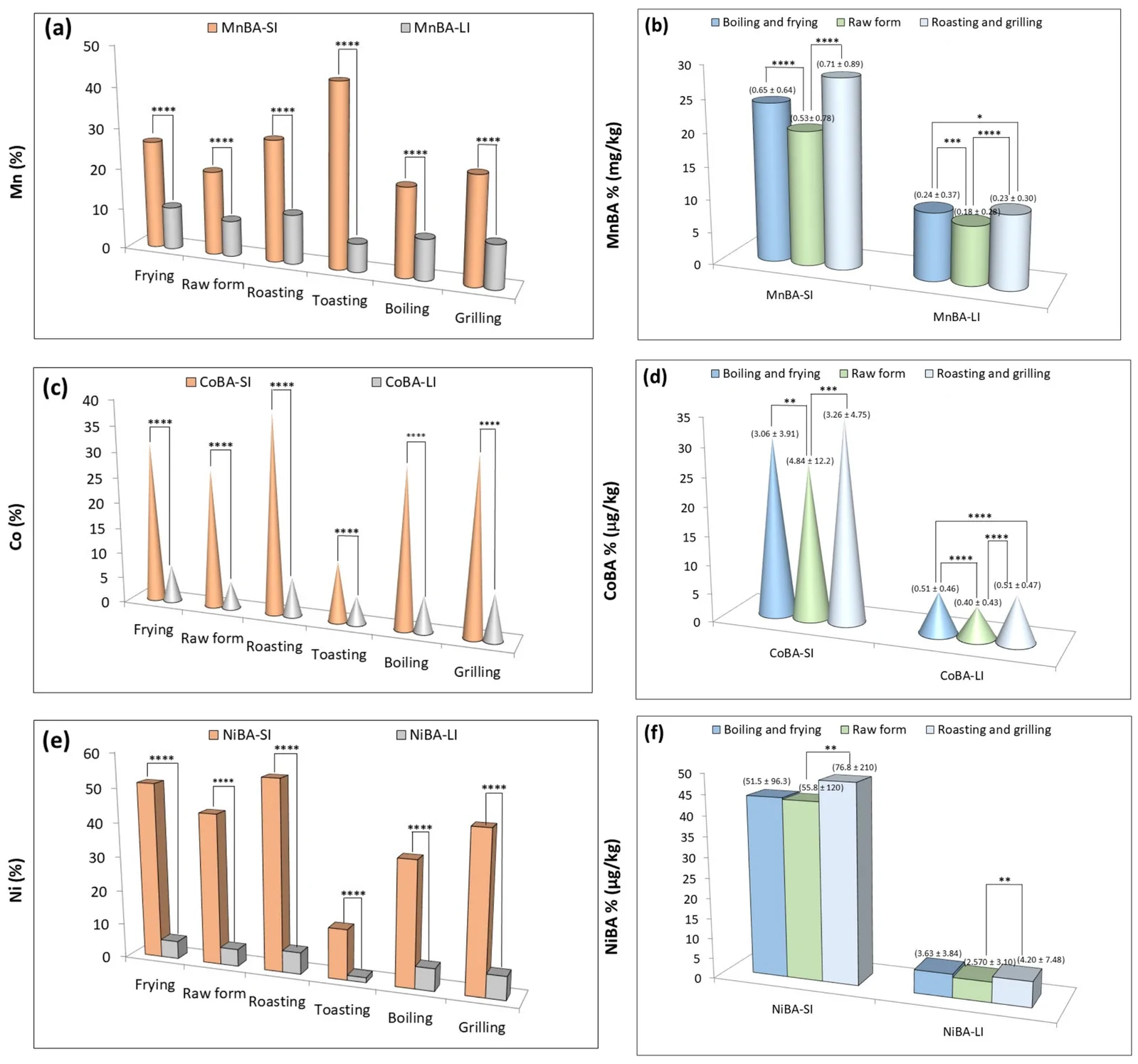
Mean bioaccessibility values (expressed as solubilized fraction available for absorption, %; and measured concentration in the soluble fraction, mg/kg or µg/kg) in the small and large intestine (BA-SI and BA-LI) of the trace metals analyzed in plant-based foods according to cooking technique: (**a**) MnBA-SI and MnBA-LI by individual cooking techniques; (**b**) MnBA-SI and MnBA-LI by grouped cooking techniques; (**c**) CoBA-SI and CoBA-LI by individual cooking techniques; (**d**) CoBA-SI and CoBA-LI by grouped cooking techniques; (**e**) NiBA-SI and NiBA-LI by individual cooking techniques; (**f**) NiBA-SI and NiBA-LI by grouped cooking techniques. Asterisks indicate statistically significant differences (* *p* < 0.05, ** *p* < 0.01, *** *p* < 0.001, **** *p* < 0.0001).

Figure 2 shows the BA of Mn, Co, and Ni in the small and large intestine in plant-based foods prepared using different household cooking techniques (Mn: Figure 2a,b; Co: Figure 2c,d; Ni: Figure 2e,f). For all three elements, bioaccessibility was significantly higher in the small intestine than in the large intestine, regardless of the cooking technique applied (*p* < 0.0001; Figure 2a,c,e; respectively). For Mn and Co, BA in the small intestine, large intestine, and the total gastrointestinal tract was significantly lower in raw plant-based foods than in those subjected to almost all other cooking techniques (Table 2; *p* < 0.05). Overall, roasting yielded the highest total BA values for all three elements (Table 2; *p* < 0.0001).

One limitation of this study is that a mass balance was not performed. Household cooking processes may concentrate minerals through moisture loss (roasting and grilling), promote their leaching into the cooking water (boiling) or oil (frying), or alter their concentrations through water or oil absorption. For Ni, BA in the small intestine (NiBA-SI), large intestine (NiBA-LI), and total gastrointestinal tract were lower in raw foods than in fried and roasted foods, whereas NiBA-LI was also lower than in boiled and grilled foods (Table 2).

The higher BA observed in cooked foods, particularly for Mn and Co, may also be related to the thermal degradation of vitamin C, which is naturally present in raw plant foods. This hypothesis warrants further investigation, as previous studies have mainly focused on the influence of vitamin C on Fe bioaccessibility [30]. In addition, thermal processing disrupts cell walls and intracellular membranes, facilitating the action of digestive enzymes and promoting the release of trace elements from the food matrix [74]. It may also increase the solubility of hydrolysis products derived from polyphenol compounds, thereby indirectly enhancing the BA of Mn, Co, and Ni. Interestingly, roasting increased the total MnBA of nuts compared with their raw counterparts, in contrast to the findings reported by others [41].

It is also noteworthy that, in plant-based foods, boiling resulted in lower total and small-intestinal bioaccessibility values for Mn, Co, and Ni than most other household cooking techniques (Table 2), probably due to mineral losses through leaching into the cooking water [75,76]. Similarly, other researchers [46] reported that parboiling significantly reduced the Ni content of rice, although its effect on NiBA was not evaluated.

For Mn, the higher BA observed in cooked plant-based foods (Table 2) may also be attributed to the degradation of anti-nutritional compounds, including polyphenols, tannins, phytic acid, oxalates, and trypsin inhibitors, as previously reported in legumes [75]. For Ni, the increase in total and small-intestinal BA in cooked foods, particularly in animal-based foods (Table 2), may also result from the release of Ni from stainless steel cookware during food preparation [44,45], although this hypothesis requires further investigation. The lower total and small-intestinal BA of Mn and Ni observed after boiling further supports the hypothesis that these elements are partially solubilized and lost into the cooking water, as previously described for parboiled rice [72].

When household cooking techniques applied to plant-based foods were grouped (boiling + frying vs. roasting + grilling) and compared with raw foods, raw samples exhibited significantly lower BA in the small intestine for Mn (MnBA-SI) and Co (CoBA-SI) (*p* < 0.05), as well as in the large intestine for Mn (MnBA-LI), and Co (CoBA-LI), (Figure 2b,d; *p* < 0.001; the corresponding box-and-whisker plots are provided in the Supplementary Material as Figure S2a,b; respectively). These findings suggest that thermal processing not only reduces the content of anti-nutritional factors but also promotes Maillard reactions, leading to the formation of compounds capable of generating soluble metal chelates, particularly during colonic fermentation [77]. Moreover, MnBA-LI and CoBA-LI were higher after roasting + grilling than after boiling + frying, indicating that more intense heat treatments enhance non-enzymatic browning and the formation of Maillard reaction products, such as furosine, methyl-furfural, and 5-hydroxymethylfurfural, which are able to form soluble chelates that maintain the solubility and BA of these trace elements [77]. Regarding Ni, its BA in both the small and large intestines was significantly lower in the raw form than in foods subjected to roasting and grilling (*p* < 0.01; Figure 2f; the corresponding box-and-whisker plots are provided in the Supplementary Material as Figure S2c).

**Figure 3:**
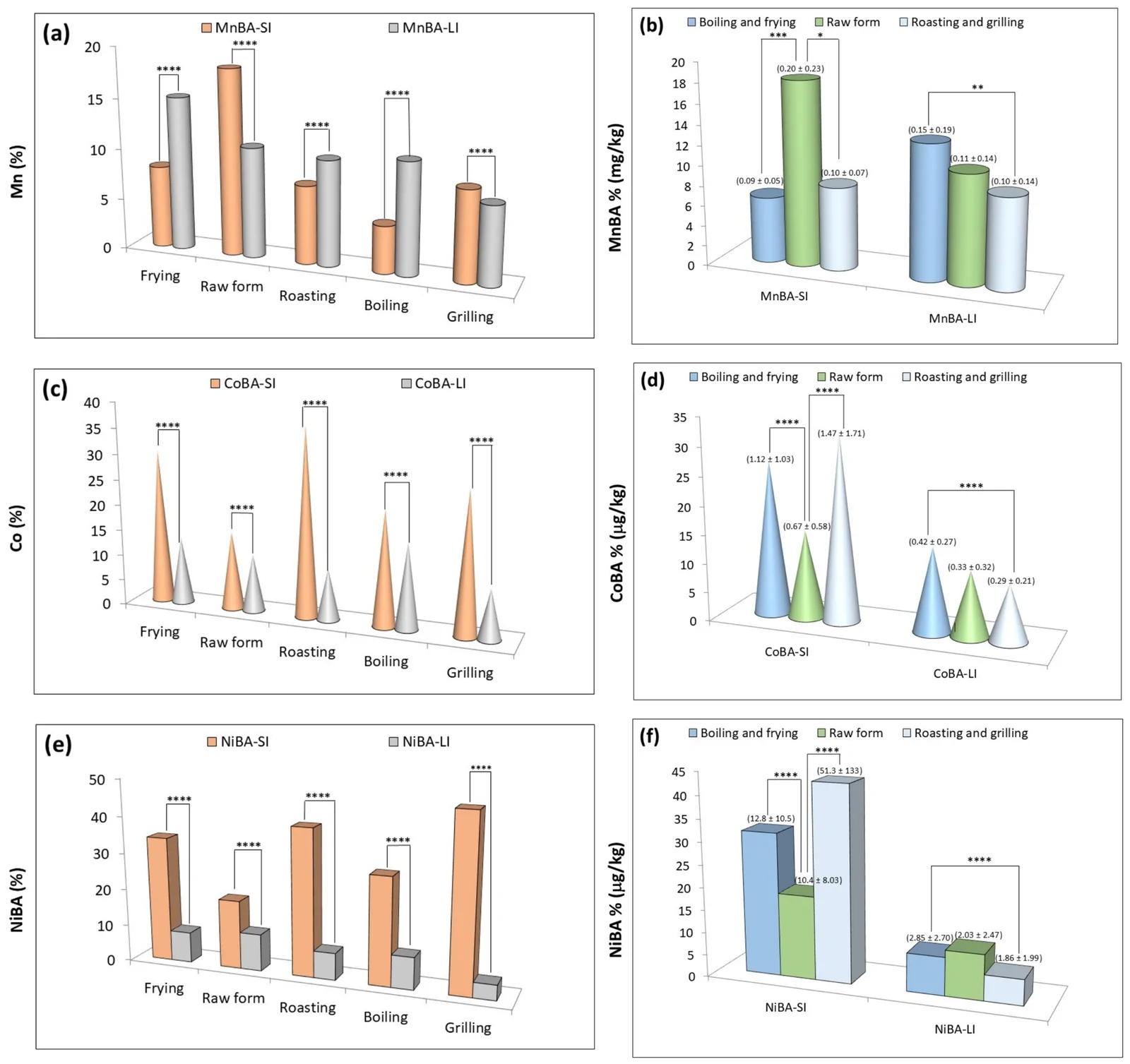
Mean bioaccessibility values (expressed as solubilized fraction available for absorption, %; and measured concentration in the soluble fraction, mg/kg or µg/kg) in the small and large intestine (BA-SI and BA-LI) of the trace metals analyzed in animal-based foods according to cooking technique: (**a**) MnBA-SI and MnBA-LI by individual cooking techniques; (**b**) MnBA-SI and MnBA-LI by grouped cooking techniques; (**c**) CoBA-SI and CoBA-LI by individual cooking techniques; (**d**) CoBA-SI and CoBA-LI by grouped cooking techniques; (**e**) NiBA-SI and NiBA-LI by individual cooking techniques; (**f**) NiBA-SI and NiBA-LI by grouped cooking techniques. Asterisks indicate statistically significant differences (* *p* < 0.05, ** *p* < 0.01, *** *p* < 0.001, **** *p* < 0.0001).

Figure 3 shows the BA of Mn, Co, and Ni in the small and large intestine in animal-based foods prepared using different household cooking techniques (Mn: Figure 3a,b; Co: Figure 3c,d; Ni: Figure 3e,f). For Co and Ni, BA was significantly higher in the small intestine than in the large intestine (Figure 3c,e) regardless of the cooking technique applied (*p* < 0.0001). Raw animal-based foods also exhibited lower total and small-intestinal BA for Co and Ni than cooked foods (Table 2; *p* < 0.05). When cooking techniques were grouped, raw foods showed significantly lower CoBA-SI than boiled + fried and roasted + grilled foods (*p* < 0.0001; Figure 3d; the corresponding box-and-whisker plots are provided in the Supplementary Material as Figure S3b) and significantly lower NiBA-SI than roasted and grilled foods combined (*p* < 0.01; Figure 3f; the corresponding box-and-whisker plots are provided in the Supplementary Material as Figure S3c).

Animal-based foods contain much lower amounts of vitamin C than plant-based foods, and this vitamin is further degraded during thermal processing. Experimental studies in rats have shown that ascorbic acid may reduce Ni accumulation in the brain by decreasing its BA [78]. Therefore, the higher Ni and Co BA observed in cooked animal-based foods may, at least in part, be associated with the degradation of vitamin C during food preparation.

In the specific case of Mn, its BA-SI in animal-based foods showed a different pattern, with significantly higher values in raw foods (Table 2), as also observed when cooking techniques were grouped (raw form vs. boiling + frying, and roasting + grilling; *p* < 0.05; Figure 3b; the corresponding box-and-whisker plots are provided in the Supplementary Material as Figure S3a). This finding may be explained by the high temperatures applied during cooking, which may induce marked protein denaturation and subsequent protein folding, thereby reducing the dialyzable fraction of Mn in the small intestine. Additionally, Mn losses through leaching during cooking cannot be ruled out.

As observed for plant-based foods, boiling also resulted in a significant reduction (*p* < 0.05) in small-intestinal BA in animal-based foods. Specifically, MnBA-SI was significantly lower than that obtained with the other cooking techniques (*p* < 0.0001; Table 2), CoBA-SI was lower than after frying and roasting (*p* < 0.0001; Table 2), and NiBA-SI was lower than after frying, roasting, and grilling (*p* < 0.0001; Table 2). This reduction may partly reflect the solubilization and leaching of these elements into the cooking water during boiling.

For Mn, large-intestinal BA exceeded small-intestinal BA in fried, roasted, and boiled animal-based foods, whereas the opposite pattern was observed in raw and grilled samples (Figure 3a). Frying, roasting, and boiling are expected to promote both vitamin C degradation and the formation of Maillard reaction products [77], which may contribute to the higher MnBA observed in the large intestine following these cooking techniques. However, this pattern was not observed in raw or grilled samples, indicating that additional factors may influence MnBA. Further studies are therefore required to elucidate the mechanisms underlying the differences among household cooking techniques.

In animal-based foods, a noteworthy finding was that large-intestinal BA following boiling + frying was significantly higher than that observed after roasting + grilling for all three elements studied (MnBA-LI, CoBA-LI, and NiBA-LI; *p* < 0.05; Figure 3b,d,f). The mechanisms underlying this pattern remain unclear and warrant further investigation.

It is well established that the effect of cooking on mineral bioaccessibility is related to the food matrix and, consequently, to the origin of the food. Therefore, matrix-dependent behavior is associated with differences in the organization and interactions of water, minerals, proteins, carbohydrates, lipids, polyphenols, and other phytochemical compounds present in plant- and animal-based foods. In plants, minerals may be compartmentalized within cell walls, starch granules, and fiber-rich networks [79]. In contrast, animal-based foods exhibit a fibrillar and myofibrillar structure combined with an emulsified architecture in which proteins and lipids are embedded within the food matrix [80]. These differences in structural organization may contribute to the distinct patterns of mineral BA between plant- and animal-based foods observed in the present study. This finding is particularly evident for Mn, whose BA in raw foods, expressed as the dialyzed and solubilized fraction available for absorption, differs markedly between plant- and animal-based foods compared with the other cooking techniques studied (Figure 2b and Figure 3b; receptively).

**Figure 4:**
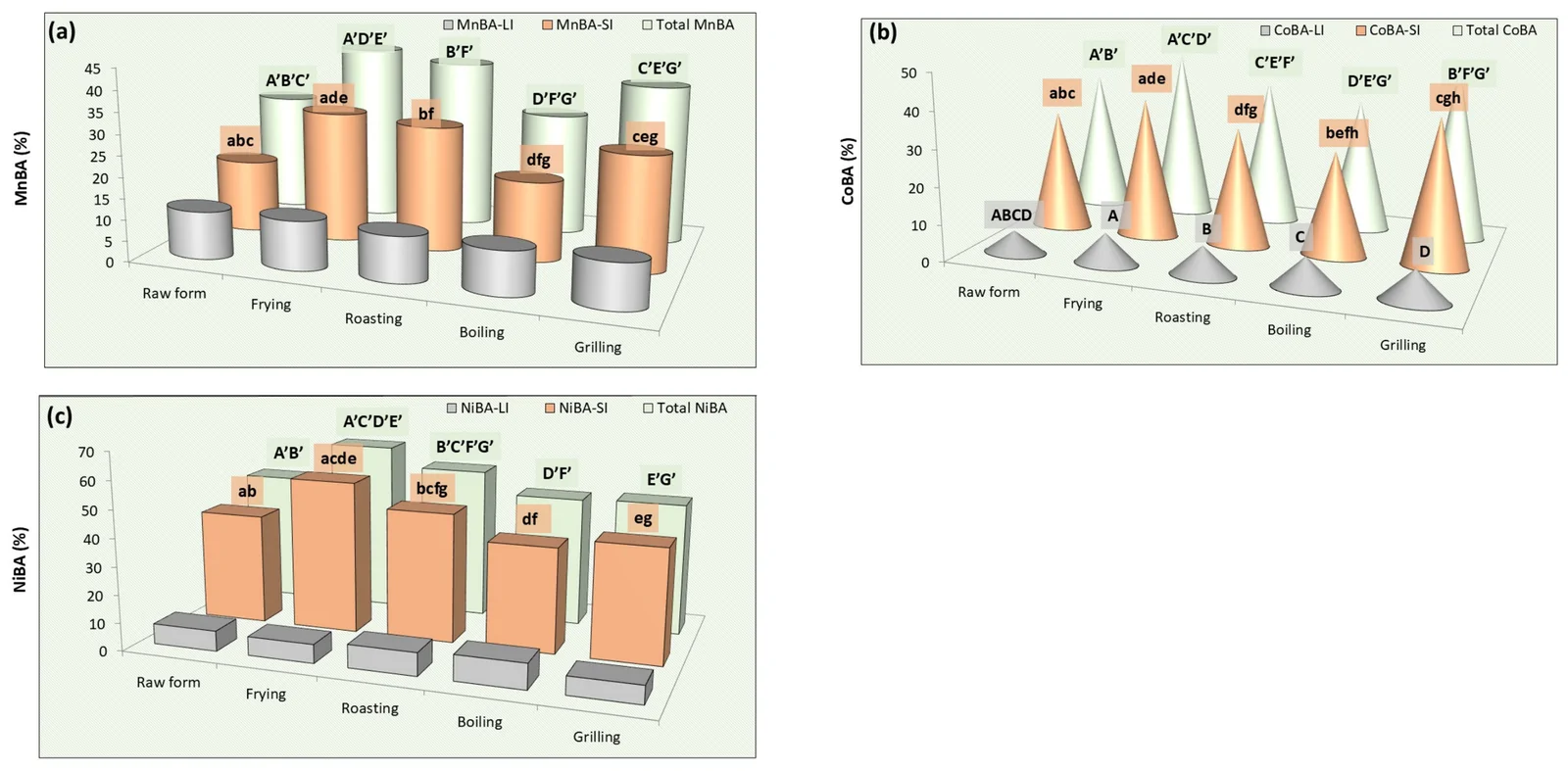
Mean total and partial bioaccessibility values (expressed as solubilized fraction available for absorption, %) of the trace metals analyzed in vegetables according to cooking technique: (**a**) Total, small-intestinal, and large-intestinal Mn bioaccessibility (Total MnBA, MnBA-SI, and MnBA-LI; respectively); (**b**) Total CoBA, CoBA-SI, and CoBA-LI; (**c**) Total NiBA, NiBA-SI, and NiBA-LI. Identical uppercase letters above the bars indicate significant differences among total and BA-LI values, whereas identical lowercase letters indicate significant differences among BA-SI values within each trace metal (*p* < 0.05).

Vegetables represented the largest food group included in the study (*n* = 59). Frying and grilling produced the highest total BA values, as well as the highest MnBA-SI and CoBA-SI values (Figure 4a,b; the corresponding box-and-whisker plots are provided in the Supplementary Material as Figure S4a,b), whereas NiBA-SI was greatest after frying and roasting (*p* < 0.0001; Figure 4c; the corresponding box-and-whisker plots are provided in the Supplementary Material as Figure S4c). In contrast, raw and boiled vegetables exhibited significantly lower small-intestinal and total BA values for all three elements (*p* < 0.05). These findings suggest that the high temperatures reached during frying, roasting, and grilling promote cell wall disruption and enhance the accessibility of digestive enzymes, thereby facilitating the release of Mn and Co from the food matrix [74]. Thermal processing may also degrade anti-nutritional compounds capable of forming insoluble complexes with these elements, further increasing their BA [75].

In the large intestine, only Co exhibited significant differences among cooking techniques, with raw vegetables showing significantly lower CoBA-LI values than all cooked forms (*p* = 0.01; Figure 4b). The higher temperatures reached during frying, roasting, and grilling may promote cell wall disruption, thereby enhancing microbial access to fermentable substrates [81]. This process is likely to increase SCFA production, facilitating the formation of soluble Co-chelates. Furthermore, these cooking techniques favor the generation of Maillard reaction products, which behave similarly to dietary fiber during colonic fermentation, stimulating SCFA production and consequently increasing CoBA [55,77].

More generally, the BA of the studied trace elements was markedly influenced by the culinary technique applied before consumption. In plant-based foods, thermal processing significantly increased the small-intestinal BA of Mn, Co, and Ni. This effect is likely attributable to the thermal disruption of the food matrix, the degradation of anti-nutritional compounds such as phytates and oxalates, and the enhanced enzymatic accessibility that promotes the release of these elements during gastrointestinal digestion. In addition, more intensive cooking methods, particularly roasting and grilling, promote Maillard reactions and the formation of low-molecular-weight compounds capable of forming soluble metal chelates. These compounds may subsequently reach the colon, where microbial fermentation generates SCFAs, lowering luminal pH and further enhancing the solubility and large-intestinal BA of Mn and Co.

At a more specific level, vegetables—the food group represented by the largest number of samples in the present study—showed the highest Mn, Co, and Ni BA following frying, grilling, and roasting. These findings suggest that, beyond their effect on mineral retention, culinary practices could substantially influence the physiological availability of trace elements by modifying both food matrix characteristics and the generation of fermentation-derived metabolites involved in intestinal mineral absorption.

### 3.3. Bioaccessibility of Mn, Co, and Ni in the Large Intestine According to Life Stage and Health Status

**Figure 5:**
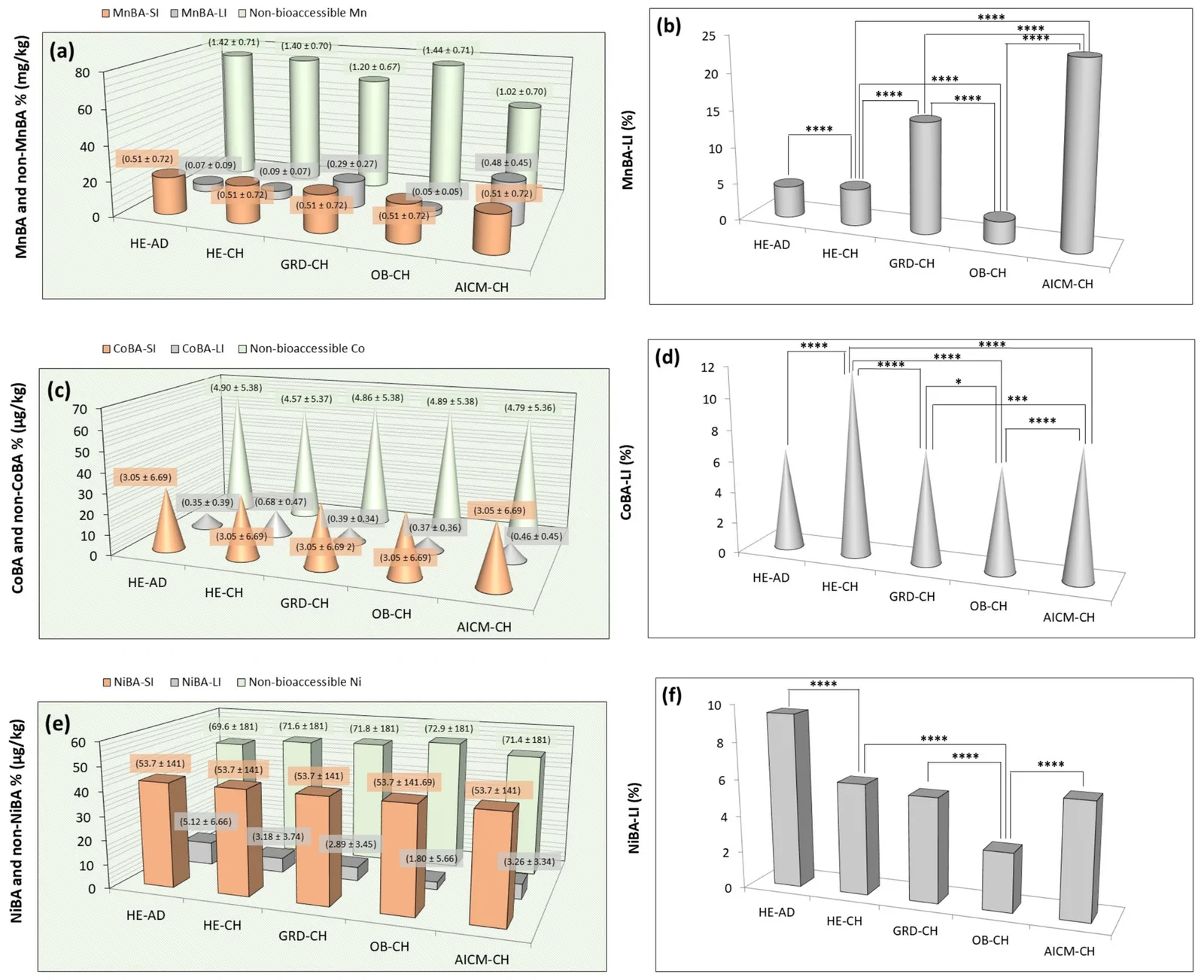
Mean bioaccessibility values (expressed as solubilized fraction available for absorption, %; and measured concentration in the soluble fraction, mg/kg or µg/kg) of the trace metals analyzed in all foodstuffs using fecal inoculum from healthy adults (HE-AD), healthy children (HE-CH), and children with gluten-related disorders (GRD-CH), with obesity (OB-CH), and allergy/intolerance to cow’s milk proteins (AICM-CH): (**a**) Bioaccessible Mn in the small intestine (MnBA-SI), large intestine (MnBA-LI), and non-bioaccessible Mn; (**b**) MnBA-LI (%); (**c**) Bioaccessible Co in the small intestine (CoBA-SI), large intestine (CoBA-LI), and non-bioaccessible Co; (**d**) CoBA-LI (%); (**e**) Bioaccessible Ni in the small intestine (NiBA-SI), large intestine (NiBA-LI), and non-bioaccessible Ni; (**f**) NiBA-LI (%). Asterisks indicate statistically significant differences (* *p* < 0.05, *** *p* < 0.001, **** *p* < 0.0001).

Figure 5 shows the distribution of the bioaccessible (Figure 5a–f) and non-bioaccessible (Figure 5a,c,e) fractions of Mn, Co, and Ni among the different study groups. Healthy children showed significantly higher MnBA-LI and CoBA-LI than healthy adults (4.93 ± 4.83 vs. 4.25 ± 6.26% and 11.9 ± 10.1 vs. 6.51 ± 8.03%, respectively; *p* < 0.0001; Figure 5b,d; the corresponding box-and-whisker plots are provided in the Supplementary Material as Figure S5a,b, respectively). These findings may reflect the greater physiological requirements for essential trace elements during growth [5,8], together with age-related differences in gut microbiota composition, given the well-established interactions between Mn homeostasis and the intestinal microbiota [62].

In contrast, healthy children showed significantly lower NiBA-LI than healthy adults (6.00 ± 7.23 vs. 9.45 ± 11.0%; *p* < 0.0001; Figure 5f; the corresponding box-and-whisker plots are provided in the Supplementary Material as Figure S5c). This finding may suggest a protective role of the pediatric gut microbiota against excessive Ni absorption. Excessive Ni exposure has been associated with impaired growth and neurodevelopment [21,82], as well as with hyperuricemia, systemic inflammation [6], and mutagenic and genotoxic effects [83]. Indeed, the gut microbiota has been proposed to act as a biological barrier that modulates the absorption and metabolism of potentially toxic elements [6]. Nevertheless, these interpretations should be considered with caution, as the relationship between the gut microbiota and exposure to trace elements such as Mn and Ni is bidirectional [6,62].

Consistent with these findings, several plant-based food groups exhibited significantly higher MnBA-LI and CoBA-LI values in healthy children than in healthy adults (*p* < 0.0001; Table 3). In contrast, the opposite trend was observed for Ni, predominantly in animal-based foods (*p* < 0.0001), further supporting a differential pattern of BA-LI according to both food origin and the trace element considered.

Among children, the pattern of large-intestinal BA differed according to the trace element considered. For Mn, children with allergy/intolerance to cow’s milk protein and those with gluten-related disorders exhibited significantly higher MnBA-LI values than healthy children (24.4 ± 20.0 and 14.8 ± 12.5 vs. 4.93 ± 4.83%, respectively; *p* < 0.0001; Figure 5b). In contrast, obese children showed significantly lower MnBA-LI than healthy children (2.92 ± 3.87 vs. 4.93 ± 4.83%; *p* < 0.0001; Figure 5b).

For Co, obese children exhibited significantly lower CoBA-LI values than HE-CH, GRD-CH, and AICM-CH (6.63 ± 8.18 vs. 11.9 ± 10.1, 7.23 ± 7.87, and 8.24 ± 7.88%, respectively; *p* = 0.05; Figure 5d). A similar pattern was observed for Ni, with OB-CH showing significantly lower NiBA-LI values than healthy children, children with gluten-related disorders, and children with allergy/intolerance to cow’s milk protein (3.16 ± 6.34 vs. 6.00 ± 7.23, 5.65 ± 7.41, and 6.18 ± 7.34%, respectively; *p* < 0.0001; Figure 5f).

These findings indicate that BA-LI is influenced by the source of the fecal inoculum used during in vitro fermentation. Compared with healthy children, OB-CH exhibited significantly lower MnBA-LI, CoBA-LI, and NiBA-LI values (*p* < 0.0001; Figure 5b,d,f, respectively). Lower Mn concentrations have previously been reported in the hair of obese women, irrespective of diabetes status [84], while reduced serum Co concentrations have also been described in obese women [85]. Moreover, Co has been suggested to exert a protective role against obesity-related metabolic disorders [13]. In contrast, Thillan et al. [86] found no differences in serum Mn or Co concentrations between primary-school children with high and normal fat intake. Although these findings are not entirely consistent, the present results suggest that the gut microbiota associated with obesity may reduce the colonic BA of these trace elements, a hypothesis that deserves further investigation.

The higher MnBA-LI values observed in AICM-CH than in HE-CH and the other pathological groups may be associated with the gut microbiota dysbiosis characteristic of IgE-mediated cow’s milk allergy, which is characterized by an increased abundance of Bacteroidota [87]. Alternatively, the elevated MnBA-LI values observed in allergy/intolerance to cow’s milk protein and gluten-related disorders may be consistent with the findings of others [7], who reported that excessive Mn exposure induces gut microbiota dysbiosis. This dysbiotic profile is characterized by an increased abundance of Bacteroidota, a reduced Bacillota/Bacteroidota ratio, elevated lipopolysaccharide production, and enterocyte toxicity accompanied by impaired intestinal barrier integrity [7]. Given that Mn absorption and retention are enhanced during childhood [8], the higher MnBA observed in AICM-CH and GRD-CH may increase their susceptibility to Mn overload and dysbiosis-related complications [7]. Consistent with this hypothesis, MnBA-LI values were significantly higher in AICM-CH and GRD-CH than in healthy children across nearly all plant-based food groups and most animal-based food groups (*p* < 0.05; Table 3). In contrast, NiBA-LI values did not differ significantly between healthy children and those with allergy/intolerance to cow’s milk protein and gluten-related disorders (Figure 5f).

For Co, healthy children exhibited significantly higher CoBA-LI values than AICM-CH and GRD-CH (*p* < 0.0001; Figure 5d). These differences may be related to the distinct large-gut microbiota profiles associated with these disorders compared with those of HE-CH, although the underlying mechanisms remain to be elucidated. Alterations in the gut microbiota have been implicated in the pathogenesis of both celiac disease [88] and cow’s milk protein allergy [89]. However, Abdukhakimova et al. [90] concluded that no consistent celiac disease-specific microbial signature can be reliably identified in either the fecal or duodenal microbiota of affected children.

As a general finding, the large-intestinal bioaccessibility of Mn, Co, and Ni differed significantly among the subject groups, highlighting the influence of both life stage (children vs. adults) and childhood disorders on the colonic BA of these trace elements. These differences are likely associated with age-dependent variations in gut microbiota composition, as well as the intestinal dysbiosis characteristic of gluten-related disorders, obesity, and allergy/intolerance to cow’s milk protein.

More specifically, healthy children exhibited significantly higher MnBA-LI and CoBA-LI values, but lower NiBA-LI values, than healthy adults. In contrast, OB-CH consistently showed the lowest BA-LI values for all three trace elements, suggesting a reduced capacity of the obese gut microbiota to promote the release and solubilization of these elements during colonic fermentation.

Among the pathological groups, AICM-CH exhibited the highest MnBA-LI values, significantly exceeding those observed in the remaining pediatric cohorts. This finding may be associated with alterations in microbial composition and fermentative activity characteristic of this condition, which could enhance the production of SCFAs and other microbial metabolites capable of increasing Mn solubilization and BA. Conversely, children with gluten-related disorders displayed intermediate BA-LI values, indicating that disease-specific dysbiosis differentially modulates trace element BA depending on the element considered.

Overall, these findings demonstrate that colonic BA of Mn, Co, and Ni is not determined solely by food composition but is also strongly influenced by host-related factors, particularly gut microbial ecology. Consequently, the nutritional value and potential toxicological impact of these trace elements should be interpreted within the context of the matrix–processing–microbiota interaction rather than from their total dietary concentrations alone.

### 3.4. Multivariate Statistical Analysis

**Figure 6:**
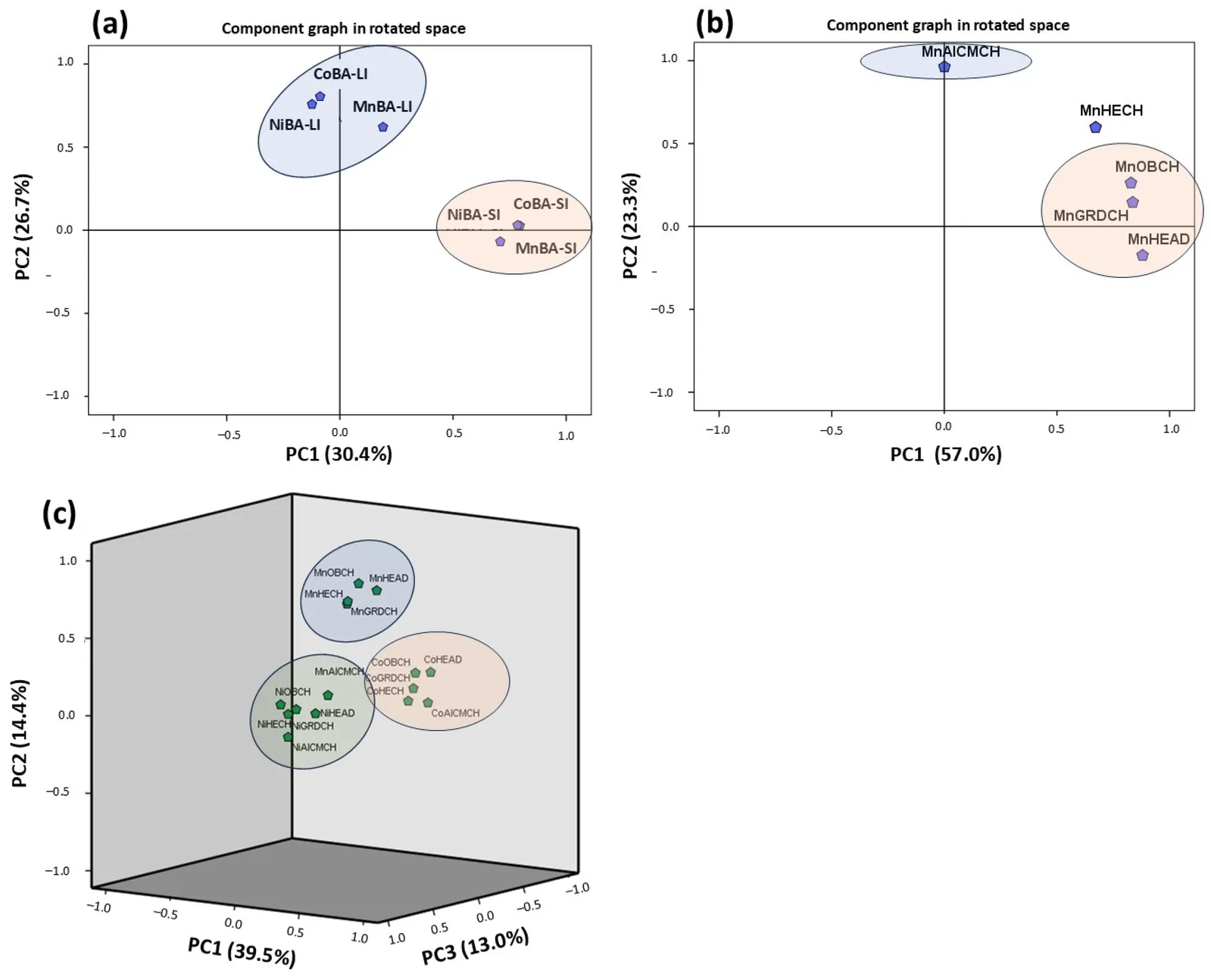
Multivariate principal component analysis (PCA). (**a**) PCA score plot showing the distribution of mean trace metal bioaccessibility values in the small and large intestines (BA-SI and BA-LI, respectively). (**b**) PCA score plot of mean large-intestinal Mn bioaccessibility (MnBA-LI) across the five fecal donor groups. (**c**) Three-dimensional loading plot of the PCA showing the relationships among large-intestinal bioaccessibility values of the trace metals analyzed across the five fecal donor groups. Data used in the PCA were raw data corresponding to the individuals (*n* = 10) included in each one of the five studied groups (HEAD: healthy adults; HECH: healthy children; GRDCH: children with gluten-related disorders; OBCH: children with obesity; and AICMCH: children with allergy/intolerance to cow’s milk proteins).

For the PCA shown in Figure 6a, the dataset included 477 and 7950 individual observations for the bioaccessibility fractions of Mn, Co, and Ni in the small and large intestines (MnBA-SI, CoBA-SI, NiBA-SI, MnBA-LI, CoBA-LI, and NiBA-LI, respectively). The mean values of these individual observations were then used to generate a dataset comprising 159 mean observations (rows) and 6 variables (columns). Two principal components explained 57.1% of the total variance. The first principal component (PC1) accounted for 30.4% of the variance and was strongly associated with MnBA-SI, CoBA-SI, and NiBA-SI, indicating that the release and solubilization of these trace metals in the small intestine are governed by similar physicochemical mechanisms. In contrast, PC2 explained 26.7% of the variance and was primarily associated with MnBA-LI, CoBA-LI, and NiBA-LI, clearly separating the colonic BA variables from those of the small intestine. This distinction indicates that trace metal BA during colonic fermentation is regulated by mechanisms different from those operating in the upper gastrointestinal tract, most likely involving microbial metabolism, SCFA production, and metal complexation.

A second PCA was conducted to evaluate the influence of the different donor groups on MnBA (Figure 6b). For this PCA, the dataset comprised 1590 individual observations (rows) and 5 variables (columns), corresponding to Mn bioaccessibility in the large intestine for the five donor groups, and a total of 7950 individual data points. The first two principal components explained 80.3% of the total variance. PC1 accounted for 57.0% of the variance and clearly differentiated MnBA-LI among healthy adults, healthy children, children with gluten-related disorders, and children with obesity. PC2 explained an additional 23.3% of the variance and distinctly separated the AICM-CH group from all remaining cohorts. This finding indicates that the AICM-CH group exhibits a unique colonic MnBA profile, probably reflecting the specific gut dysbiosis associated with this pathological condition.

Finally, a third PCA using Varimax rotation with Kaiser normalization was performed to evaluate the relationships among the large-intestinal BA values of Mn, Co, and Ni across the five donor groups (Figure 6c). For this PCA, the dataset comprised 1590 individual observations (rows) and 15 variables (columns), corresponding to Mn, Co, and Ni bioaccessibility in the large intestine across the five fecal donor groups studied, and a total of 23,850 individual data points. Five principal components with eigenvalues > 1 (5.92, 2.16, 1.95, 1.17, and 1.08) explained 81.8% of the total variance. PC1 accounted for 39.5% of the variance and was dominated by CoBA-LI across all donor groups, together with MnBA-LI in GRD-CH, OB-CH, and HE-AD, and NiBA-LI in GRD-CH and OB-CH. PC2 explained 14.4% of the variance and was mainly associated with NiBA-LI in healthy children and adults, whereas PC3 accounted for 13.0% of the variance and was characterized primarily by MnBA-LI in the AICM-CH and HE-CH groups.

The three-dimensional loading plot (Figure 6c) revealed three well-defined clusters. The first comprised MnBA-LI values from HE-AD, HE-CH, OB-CH, and GRD-CH, indicating similar colonic MnBA patterns among these cohorts. The second cluster included CoBA-LI values from all donor groups, suggesting a common microbial response governing Co release during fermentation. The third cluster consisted of all NiBA-LI values together with the MnBA-LI values of the AICM-CH group, highlighting the distinctive fermentation behavior of children with allergy/intolerance to cow’s milk protein. Collectively, these multivariate analyses demonstrate that colonic BA of Mn, Co, and Ni is strongly influenced by donor microbiota composition and that pathology-associated dysbiosis, particularly in AICM-CH subjects, produces characteristic trace metal bioaccessibility patterns.

### 3.5. Mn, Co, and Ni Intake per Serving and Risk Assessment

Regarding dietary intake per serving, the highest Mn contents (>0.40 mg/serving) were found in spinach, eggplant, onion, and yogurt; the highest Co contents (>2 µg/serving) in whole meal penne, beer, cauliflower, and diet cola; and the highest Ni contents (>30 µg/serving) in spinach, lentils, rice, bread, and apple. Based on these values, the percentages of the DRIs for Mn (2.3, 1.8, and 1.5 mg/day for healthy adult men, healthy adult women, and children aged 3–8 years, respectively; [59]; Table 4) ranged from 0.20–27.7%, 0.25–35.4%, and 0.30–42.5% per serving, respectively, with sunflower oil and spinach representing the lowest and highest contributors. As the TUILs for Mn are 11 mg/day for adults and 3 mg/day for children aged 4–8 years [59], the estimated Mn intake per serving does not pose a toxicological concern for either population group.

Although the cumulative intake from multiple daily servings would be unlikely to exceed the established TUILs for Ni, particular attention should be paid to children with allergy/intolerance to cow’s milk protein and gluten-related disorders, who exhibited higher total MnBA (59.6% and 35.4%, respectively) than healthy children, mainly due to their increased MnBA-LI. Therefore, periodic monitoring of Mn intake from foods providing relatively high amounts per serving, such as onion and yogurt, particularly when combined with elevated MnBA, may be advisable in AICM-CH and GRD-CH.

For Co, the percentages of the RfD established by the U.S. EPA (21 µg/day for a 70-kg adult and 5.4–8.1 µg/day for children weighing 18–27 kg; [10]) ranged from 0.18–11.7% for adults and 0.46–45.5% for children, with sunflower oil and coke representing the lowest and highest contributors, respectively (Table 4). However, considering the RfD of 0.3 µg Co/kg body weight/day [89], the estimated Co intake per serving, and even the total daily dietary intake, should be monitored mainly in young children. In this sense, for some foods (whole grain penne, cauliflower, beer and coke), the estimated coverage percentage for RfD for Co is higher than 40% for children aged 4 y (Table 4). Consequently, long-term monitoring of Co contents and BA levels in foods would be necessary to prevent potential toxicological health risks.

For Ni, the TUILs (1000 µg/day for healthy adults and 300 µg/day for children aged 4–8 years; [4]) ranged from 0.03–7.91% and 0.11–26.4% per serving, respectively, with sunflower oil and spinach representing the lowest and highest contributors (Table 4). Although a daily Ni intake of 25–35 µg has been suggested, typical dietary intakes generally exceed 70 µg/day [4]. In addition to spinach, lentils, rice, bread, and apples, which each provide more than 30 µg Ni per serving, foods supplying more than 20 µg per serving, including diet cola, zucchini, and mixed nuts, should also be considered important dietary sources. Considering the combined consumption of several servings from some food groups evaluated in this study, total daily Ni intake is likely to exceed 70 µg/day. Therefore, given the potential toxicity of Ni, dietary exposure should continue to be monitored, with particular attention to both raw and cooked foods and to their corresponding bioaccessibility values.

Despite all it should be kept in mind that the concentration reported after digestion and fermentation in this study is the measured concentration of each element (Mn, Co, and Ni) in the soluble fraction.

Table S8 summarizes previous studies evaluating the daily dietary intake of Mn, Co, and Ni from foods also included in the present study, based on defined serving sizes. These studies also assessed the potential carcinogenic and non-carcinogenic health risks associated with dietary exposure in both healthy adults and children [15,16,21,22,24,25,42,45,46,66,67,72,73,91,92,93,94,95].

In the case of Mn, most of the studies summarized in Table S8 did not report any health risk associated with the daily consumption of the specified serving size, even when considering the overall dietary intake from other foods consumed throughout the day. However, two studies identified a potential health risk associated with the consumption of Nigerian [46] and Malaysian rice [72]. Nevertheless, both studies concluded that hydrothermal parboiling treatment substantially reduced the potential health risk associated with Mn exposure. A recent study by Stolecka et al. [15], which evaluated vegetables, fruits, and cereals consumed in Poland, concluded that the estimated daily Mn intake exceeded the TUIL, representing a significant health risk. These findings are consistent with the results of the present study, in which certain food servings were identified as major contributors to Mn intake and may pose a potential concern for children, particularly those with allergy/intolerance to cow’s milk protein, owing to their higher MnBA (measured as the percentage of the total amount of Mn that remained soluble following the in vitro procedure applied in the study).

Regarding Co, the number of available studies is more limited. Most of those summarized in Table S8 also reported a low potential health risk for both children and adults [24,89,91]. However, Stolecka et al. [15] estimated daily Co intakes exceeding the typical range of 5–8 µg/day and suggested a potential non-carcinogenic health risk when high BA values are assumed. These findings highlight the importance of assessing the BA of potentially toxic elements, in addition to their total concentrations in foods, as demonstrated in the present study.

Finally, several of the studies summarized in Table S8 assessing the potential health risks associated with Ni exposure from individual food servings or estimated daily dietary intake concluded that Ni intake does not pose a significant health risk [24,25,42,91,94]. However, others [16] reported that cooking in stainless steel cookware substantially increased the Ni content of foods owing to metal leaching, highlighting the need for continued monitoring. These authors estimated very high Ni intakes following the consumption of 200 g servings of soy yogurt and tofu prepared under these conditions. On the other hand, Ortiz et al. [45] reported that although cooking rice in stainless steel cookware increases its Ni concentration, the BA of the leached Ni is very low and therefore does not represent a health concern.

In contrast, several studies have highlighted the potential chronic toxicological risk associated with dietary Ni exposure [21,66,72,92,94]. Therefore, given that Ni is a potentially toxic trace element, its concentration in foods, intake per serving and through the total daily diet, together with its bioaccessible fraction, should be routinely monitored to prevent potential adverse health effects, particularly in children, who are more susceptible to Ni exposure.

Finally, it is well established that the food matrix can markedly influence the BA of the elements under investigation. However, the present study did not investigate how the food matrix may affect the BA of different supplements containing the elements evaluated. In this regard, over the past 30 years, the consumption of nutritional supplements, particularly multi-micronutrient supplements, has increased among populations in Western countries, mainly to correct potential nutritional deficiencies of various elements resulting from unbalanced dietary patterns [96].

Regarding the specific elements analyzed in the present study, only two are essential trace elements, namely Mn and Co. In addition, research efforts are being undertaken to develop strategies for supplementing diets and commonly consumed foods with essential elements, particularly Fe and Zn, for which nutritional deficiencies are among the most frequently reported in the population [97,98].

In the present research, we observed that, irrespective of the amount of Mn, Co, and Ni present in the foods analyzed, the fraction solubilized following the in vitro digestion/fermentation process was highly variable. Therefore, future studies should investigate the in vitro BA of different mineral salts commonly used in nutritional supplements containing Mn and Co, when consumed or added to different foods prepared and consumed under typical conditions. Such studies would allow the effect of the food matrix on the BA of these elements to be assessed.

This approach could also determine whether the consumption of supplements together with foods, either simultaneously or separately, affects the resulting BA of these elements. Ultimately, these studies could contribute to establishing appropriate consumption guidelines for nutritional supplements containing Mn and Co for the general population.

## 4. Conclusions

First, it should be noted that the concentration reported for each bioaccessible fraction after digestion and fermentation is the measured concentration of the element in the soluble fraction, whereas bioaccessibility is expressed as the percentage of the total amount of the element that remained soluble following the in vitro procedure applied in the study.

This study demonstrates that the BA of Mn, Co, and Ni is determined by a complex interaction between food matrix, culinary processing, and the gastrointestinal environment. Plant-based foods generally exhibited higher total BA of Mn and Ni than animal-based foods, while colonic fermentation made a relevant contribution to the bioaccessible fraction of all three metals, highlighting the role of the gut microbiota in trace metal availability

Culinary processing significantly modified metal BA. In general, cooking enhanced the BA of Mn, Co, and Ni compared with raw foods to a major extent in plant-based foods, although the magnitude of this effect depended on the cooking technique and the plant- and animal-based food matrix. These findings indicate that food preparation should be considered when assessing dietary exposure to both essential and potentially toxic trace metals.

Life stage and childhood health status also influenced metal BA. Healthy children showed greater colonic BA of Mn and Co than adults, whereas children with cow’s milk protein allergy and gluten-related disorders exhibited markedly increased MnBA, suggesting that disease-associated alterations of the gut microbiota may modify intestinal metal availability. Although estimated per-serving intakes of Mn and Co did not indicate a toxicological concern under typical dietary conditions, several commonly consumed foods provided relatively high Ni intakes, particularly for children, warranting continued dietary monitoring.

Overall, these findings provide new evidence that evaluating dietary exposure to trace metals should consider not only their total concentration but also their BA after digestion and fermentation. Future studies should validate these observations in vivo, deeply investigate the microbial mechanisms regulating trace metal BA, and evaluate long-term dietary exposure in vulnerable populations, particularly children with gastrointestinal or allergic disorders.

## Figures and Tables

**Table 1:** Mean Mn, Co and Ni content, bioaccessibility values in the small and large intestine (MnBA-SI/MnBA-LI, CoBA-SI/CoBA-LI, and NiBA-SI/NiBA-LI; expressed as the solubilized fraction available for absorption) in different foodstuffs.

Food Group	Mn	MnBA-SI		MnBA-LI		Total MnBA	
	(mg/kg)	(%) ^#†^	(mg/kg)	(%) ^#†^	(mg/kg)	(%) ^#†^	(mg/kg)
***Plant foods***							
Nuts	3.48 ± 1.76	53.9 ± 9.84 ^ABCDEF^	1.94 ± 0.76	15.6 ± 15.5 ^ABCDE^	0.57 ± 0.63	69.5 ± 19.3 ^ABCDEF^	2.52 ± 1.12
Cereals	3.16 ± 1.43	30.3 ± 17.8 ^AGHIJK^	0.93 ± 0.60	7.85 ± 10.7 ^AFGHIJK^	0.20 ± 0.22	38.2 ± 20.2 ^AGHIJK^	1.13 ± 0.65
Fruits	1.76 ± 0.49	16.9 ± 9.36 ^BGLMNOP^	0.28 ± 0.15	11.2 ± 13.6 ^BFLM^	0.19 ± 0.23	28.1 ± 16.7 ^BGLMNOP^	0.47 ± 0.27
Vegetables	2.24 ± 0.99	24.9 ± 15.0 ^DMQRST^	0.63 ± 0.78	10.9 ± 13.8 ^CGNO^	0.23 ± 0.32	35.8 ± 20.3 ^CLQRST^	0.86 ± 0.90
Legumes	1.83 ± 0.39	28.7 ± 10.0 ^DMQUVW^	0.54 ± 0.25	13.1 ± 15.4 ^HPQ^	0.23 ± 0.26	41.7 ± 17.8 ^DHMQUVW^	0.76 ± 0.35
Oils	0.45 ± 0.16	6.81 ± 8.92 ^EINRUX^	0.04 ± 0.06	0.55 ± 0.93 ^DILNPRS^	0.01 ± 0.01	7.36 ± 8.88 ^EINRUX^	0.04 ± 0.05
Beverages + infusions	1.02 ± 0.35	6.88 ± 6.88 ^FJOSVY^	0.06 ± 0.07	0.23 ± 0.34 ^EJMOQRT^	0.01 ± 0.01	7.11 ± 6.92 ^FJOSVY^	0.06 ± 0.07
Others	3.33 ± 1.21	65.7 ± 19.9 ^KPTWXY^	2.42 ± 1.57	9.69 ± 9.17 ^KST^	0.30 ± 0.27	75.4 ± 20.1 ^KPTWXY^	2.72 ± 1.53
*All plant foods*	*2.20 ± 1.19*	*24.7 ± 17.8*	*0.64 ± 0.78*	*10.1 ± 13.5*	*0.22 ± 0.32*	*34.8 ± 21.1*	*0.85 ± 0.91*
***Animal foods***							
Dairy products	1.25 ± 0.39	15.2 ± 17.6 ^abcdef^	0.16 ± 0.19	14.5 ± 19.7 ^abc^	0.13 ± 0.16	29.7 ± 30.4 ^ab^	0.30 ± 0.28
Chicken	1.21 ± 0.23	5.92 ± 3.74 ^aghij^	0.07 ± 0.04	9.85 ± 13.9 ^d^	0.12 ± 0.19	15.8 ± 14.0 ^cd^	0.19 ± 0.19
Beef	1.50 ± 0.23	7.13 ± 2.42 ^bklmn^	0.10 ± 0.03	10.1 ± 14.1 ^efgh^	0.15 ± 0.22	17.2 ± 14.1 ^ef^	0.26 ± 0.22
Salmon	1.46 ± 0.33	7.96 ± 2.69 ^cgopqr^	0.12 ± 0.08	6.90 ± 11.1 ^eij^	0.09 ± 0.14	14.9 ± 10.9 ^gh^	0.22 ± 0.15
Cod	1.68 ± 0.39	5.05 ± 0.78 ^dhkos^	0.09 ± 0.03	5.87 ± 9.42 ^afkl^	0.09 ± 0.14	10.9 ± 9.57 ^acegijk^	0.18 ± 0.15
Egg	1.66 ± 0.50	11.9 ± 7.70 ^ilpstu^	0.16 ± 0.08	5.65 ± 8.59 ^bgmn^	0.09 ± 0.14	17.5 ± 11.7 ^il^	0.26 ± 0.16
Lamb	0.90 ± 0.21	6.97 ± 5.78 ^emqt^	0.06 ± 0.06	20.5 ± 21.4 ^cdhikmo^	0.17 ± 0.17	27.5 ± 23.0 ^dfhjlm^	0.23 ± 0.18
Pork	1.18 ± 0.31	4.79 ± 2.66 ^fjnru^	0.05 ± 0.02	11.4 ± 13.9 ^jlno^	0.13 ± 0.15	16.1 ± 14.6 ^bkm^	0.17 ± 0.16
*All animal foods*	*1.35 ± 0.41*	*8.78 ± 10.5*	*0.11 ± 0.11*	*10.9 ± 15.8*	*0.12 ± 0.17*	*19.8 ± 20.1*	*0.23 ± 0.21*
**Food group**	**Co**	**CoBA-SI**		**CoBA-LI**		**Total CoBA**	
	**(** **µ** **g/kg)**	**(%) ^#†^**	**(µg/kg)**	**(%) ^#†^**	**(µg/kg)**	**(%) ^#†^**	**(µg/kg)**
***Plant foods***							
Nuts	17.1 ± 10.0	56.3 ± 19.3 ^ABCDEFG^	10.8 ± 8.49	6.30 ± 5.06 ^ABCDEF^	0.82 ± 0.54	62.6 ± 18.0 ^ABCDEFG^	11.7 ± 8.42
Cereals	16.3 ± 13.3	20.4 ± 19.5 ^AHIJKL^	2.71 ± 2.90	3.48 ± 4.81 ^AGHIJK^	0.41 ± 0.60	23.8 ± 20.0 ^AHIJKLM^	3.12 ± 2.82
Fruits	5.76 ± 3.26	22.7 ± 19.4 ^BHMNOPQ^	1.59 ± 3.05	9.89 ± 7.74 ^BGLMNOP^	0.47 ± 0.36	32.6 ± 20.7 ^BHNOPQR^	2.07 ± 3.07
Vegetables	7.67 ± 3.65	34.3 ± 16.5 ^CIMRSTU^	2.51 ± 1.77	7.83 ± 6.44 ^CHLQRS^	0.53 ± 0.42	42.2 ± 18.2 ^CINSTUV^	3.05 ± 1.89
Legumes	10.8 ± 8.78	42.1 ± 19.4 ^DJNRVWX^	5.87 ± 7.73	6.85 ± 6.11 ^IMTUV^	0.51 ± 0.36	48.9 ± 18.6 ^DJOSWXY^	6.37 ± 7.64
Oils	4.09 ± 2.09	11.7 ± 16.1 ^EKOSVYZ^	0.62 ± 0.97	0.16 ± 0.16 ^DJNQTWX^	0.01 ± 0.00	11.9 ± 16.0 ^EKPTWZA’^	0.63 ± 0.94
Beverages + infusions	9.08 ± 4.01	19.4 ± 16.0 ^FPTWYA’^	2.14 ± 2.31	0.07 ± 0.07 ^EKORUWY^	0.01 ± 0.01	19.5 ± 16.0 ^FLQUXZB’^	2.15 ± 2.25
Others	56.5 ± 12.4	89.4 ± 7.57 ^GLQUXZA’^	51.4 ± 16.6	1.69 ± 0.85 ^FPSVXY^	1.02 ± 0.65	91.1 ± 8.12 ^GMRVYA’B’^	52.4 ± 15.9
*All plant foods*	*9.63 ± 9.52*	*31.2 ± 21.3*	*3.64 ± 7.53*	*6.89 ± 6.77*	*0.50 ± 0.59*	*38.1 ± 22.5*	*4.13 ± 7.66*
***Animal foods***							
Dairy products	4.26 ± 2.42	14.6 ± 14.5 ^abcdefg^	0.91 ± 1.42	11.6 ± 12 ^abc^	0.37 ± 0.31	26.2 ± 17.2 ^abcde^	1.29 ± 1.42
Chicken	2.63 ± 1.20	35.4 ± 33.2 ^ahijklm^	0.93 ± 0.70	13.9 ± 13.3 ^defg^	0.29 ± 0.21	49.1 ± 31.2 ^afg^	1.22 ± 0.68
Beef	4.03 ± 1.00	18.0 ± 4.38 ^bhnop^	0.71 ± 0.22	10.1 ± 6.96 ^hij^	0.39 ± 0.25	28.1 ± 7.46 ^fhijk^	1.09 ± 0.28
Salmon	5.75 ± 1.74	41.5 ± 16.0 ^cinqrs^	2.33 ± 1.28	6.00 ± 5.85 ^adhklm^	0.28 ± 0.24	47.5 ± 18.2 ^bhlm^	2.62 ± 1.25
Cod	4.26 ± 1.29	37.0 ± 9.75 ^djoqtu^	1.46 ± 0.12	5.89 ± 3.82 ^beino^	0.25 ± 0.19	42.9 ± 10.3 ^cino^	1.71 ± 0.21
Egg	4.20 ± 2.34	21.8 ± 10.7 ^ekrtv^	0.89 ± 0.62	8.26 ± 6.99 ^fkpq^	0.27 ± 0.20	30.1 ± 11.5 ^glnpq^	1.16 ± 0.65
Lamb	2.31 ± 0.83	19.8 ± 16.3 ^flsuw^	0.41 ± 0.27	27.0 ± 15.8 ^cgjlnpr^	0.55 ± 0.26	46.8 ± 28.0 ^djp^	0.96 ± 0.42
Pork	4.11 ± 2.54	41.5 ± 27.0 ^gmpvw^	2.05 ± 2.63	15.6 ± 14.7 ^moqr^	0.39 ± 0.24	57.2 ± 28.8 ^ekmoq^	2.44 ± 2.56
*All animal foods*	*4.03 ± 2.09*	*27.5 ± 21.2*	*1.21 ± 1.35*	*12.1 ± 12.4*	*0.36 ± 0.37*	*39.6 ± 23.1*	*1.58 ± 1.36*
**Food group**	**Ni**	**NiBA-SI**		**NiBA-LI**		**Total NiBA**	
	**(** **µ** **g/kg)**	**(%) ^#†^**	**(µg/kg)**	**(%) ^#†^**	**(µg/kg)**	**(%) ^#†^**	**(µg/kg)**
***Plant foods***							
Nuts	509 ± 335	71.8 ± 11.4 ^ABCDEF^	359 ± 241	1.77 ± 1.67 ^ABCDEF^	6.19 ± 4.35	73.5 ± 11.5 ^ABCDEF^	366 ± 237
Cereals	289 ± 431	30.4 ± 29.5 ^AGHIJKL^	47.9 ± 75.0	2.34 ± 3.94 ^AGHIJK^	2.26 ± 2.98	32.7 ± 30.9 ^AGHIJKL^	50.2 ± 73.7
Fruits	67.5 ± 74.2	51.8 ± 29.1 ^BGMNOP^	20.5 ± 14.1	7.37 ± 7.77 ^BGLMNO^	3.13 ± 3.66	59.2 ± 30.5 ^BGMNO^	23.7 ± 14.5
Vegetables	97.6 ± 177	44.0 ± 24.9 ^CHMQRST^	28.5 ± 23.7	7.56 ± 8.36 ^CHPQRS^	4.25 ± 6.68	51.6 ± 25.8 ^CHMPQRS^	32.7 ± 24.7
Legumes	327 ± 481	56.1 ± 22.8 ^DIQUV^	262 ± 474	2.76 ± 3.39 ^ILPTUV^	3.43 ± 3.99	58.9 ± 22.2 ^DIPTU^	265 ± 464
Oils	51.2 ± 28.2	35.1 ± 14.7 ^EJNRUW^	18.1 ± 16.5	0.08 ± 0.09 ^DJMQTW^	0.03 ± 0.03	35.2 ± 14.7 ^EJNQTW^	18.2 ± 15.9
Beverages + infusions	63.0 ± 27.4	37.0 ± 22.5 ^FKOSVX^	21.4 ± 16.5	0.07 ± 0.15 ^EKNRUX^	0.04 ± 0.09	37.1 ± 22.5 ^FKORUV^	21.4 ± 16.1
Others	614 ± 71.4	66.8 ± 31.0 ^LPTWX^	389 ± 154	1.10 ± 0.91 ^FOSVWX^	6.32 ± 4.82	67.9 ± 31.7 ^LSVW^	395 ± 146
*All plant foods*	*150 ± 267*	*45.8 ± 27.2*	*62.1 ± 154*	*5.68 ± 7.50*	*3.53 ± 5.38*	*51.4 ± 28.3*	*65.6 ± 154*
***Animal foods***							
Dairy products	47.9 ± 29.2	20.1 ± 16.4 ^abcdef^	12.1 ± 14.2	9.02 ± 14.0 ^ab^	2.51 ± 2.74	29.2 ± 16.3 ^abcdefg^	14.7 ± 14.4
Chicken	36.8 ± 8.29	48.6 ± 36.8 ^agh^	17.6 ± 12.7	6.06 ± 5.31 ^cd^	2.19 ± 2.08	54.7 ± 36.3 ^ahi^	19.3 ± 12.5
Beef	167 ± 241	45.8 ± 29.3 ^bijkl^	146 ± 249	8.49 ± 10.2 ^ef^	2.83 ± 2.38	54.3 ± 25.9 ^bjk^	149 ± 240
Salmon	55.0 ± 32.5	51.4 ± 19.8 ^cimno^	28.7 ± 23.4	7.54 ± 10.2 ^g^	1.98 ± 1.76	58.9 ± 22.3 ^clmn^	30.6 ± 22.2
Cod	56.9 ± 8.96	18.1 ± 6.09 ^gjmpqr^	10.8 ± 5.32	3.33 ± 3.23 ^acehi^	1.88 ± 1.85	21.5 ± 6.14 ^dhjlop^	12.6 ± 5.25
Egg	65.8 ± 35.7	14.3 ± 7.64 ^dhknpst^	7.31 ± 3.46	4.10 ± 6.81 ^bdfjk^	2.05 ± 3.52	18.4 ± 10.5 ^eikmqr^	9.36 ± 4.71
Lamb	34.1 ± 15.4	40.0 ± 7.67 ^eloqsu^	12.6 ± 4.42	10.9 ± 12.3 ^ghj^	2.70 ± 2.27	50.9 ± 17.6 ^fnoqs^	15.3 ± 4.93
Pork	29.9 ± 10.5	51.3 ± 18.9 ^frtu^	15.5 ± 9.03	8.41 ± 8.30 ^ik^	2.23 ± 2.12	59.7 ± 22.0 ^gprs^	17.7 ± 9.50
*All animal foods*	*60.0 ± 90.7*	*34.9 ± 250*	*29.1 ± 89.6*	*7.43 ± 10.3*	*2.31 ± 2.43*	*42.3 ± 26.3*	*31.4 ± 89.3*

^#^ Values are presented as mean ± SD (fresh weight basis). Identical uppercase superscript letters within columns for bioaccessibility values of each trace metal analyzed indicate statistically significant differences among different categories of plant-based foods on the Kruskal–Wallis and Mann–Whitney U tests (*p* < 0.05). ^†^ Values are presented as mean ± SD (fresh weight basis). Identical lowercase superscript letters within columns for bioaccessibility values of each trace metal analyzed indicate statistically significant differences among different categories of animal-based foods on the Kruskal–Wallis and Mann–Whitney U tests (*p* < 0.05).

**Table 2:** Mean Mn, Co, and Ni concentrations (fresh weight) and bioaccessibility values in the small and large intestine (MnBA-SI/MnBA-LI, CoBA-SI/CoBA-LI, and NiBA-SI/NiBA-LI, respectively; expressed as solubilized fraction available for absorption) in plant- and animal-based foods according to household cooking techniques.

Cooking Techniques	Mn	MnBA-SI		MnBA-LI		Total MnBA	
	(mg/kg)	(%) ^#†^	(mg/kg)	(%) ^#†^	(mg/kg)	(%) ^#†^	(mg/kg)
***Plant foods***							
Frying	2.79 ± 1.60	26.4 ± 14.2 ^ABC^	0.83 ± 0.78	10.4 ± 14.4 ^AB^	0.28 ± 0.44	36.8 ± 20.6 ^ABCD^	1.12 ± 1.00
Raw form	1.96 ± 1.16	20.4 ± 19.5 ^ADEFG^	0.53 ± 0.78	8.71 ± 12.9 ^AC^	0.18 ± 0.28	29.0 ± 24.4 ^AEFG^	0.70 ± 0.90
Roasting	2.28 ± 1.30	29.5 ± 19.1 ^DHI^	0.83 ± 1.14	12.0 ± 14.1 ^BD^	0.25 ± 0.34	41.5 ± 23.8 ^BEHIJ^	1.08 ± 1.26
Toasting	3.11 ± 0.85	44.2 ± 3.52 ^BEHJK^	1.35 ± 0.29	6.85 ± 6.82 ^C^	0.21 ± 0.22	51.0 ± 7.85 ^CFIKL^	1.55 ± 0.38
Boiling	2.03 ± 0.64	21.5 ± 12.0 ^CFIJL^	0.43 ± 0.28	9.87 ± 12.8 ^D^	0.18 ± 0.24	31.3 ± 18.6 ^DJLM^	0.61 ± 0.39
Grilling	1.95 ± 0.61	25.9 ± 15.6 ^GKL^	0.53 ± 0.50	10.5 ± 12.7	0.21 ± 0.26	36.4 ± 21.3 ^GKM^	0.73 ± 0.60
***Animal foods***							
Frying	1.35 ± 0.49	8.07 ± 4.08 ^ab^	0.10 ± 0.05	15.1 ± 19.9 ^abc^	0.16 ± 0.20	23.2 ± 21.9 ^a^	0.26 ± 0.20
Raw form	1.32 ± 0.25	18.3 ± 21.6 ^acde^	0.20 ± 0.23	9.47 ± 12.6 ^ad^	0.11 ± 0.14	27.8 ± 31.8	0.32 ± 0.33
Roasting	1.39 ± 0.56	7.64 ± 2.89 ^cf^	0.10 ± 0.07	10.3 ± 16.3 ^b^	0.10 ± 0.14	18.0 ± 16.8	0.20 ± 0.15
Boiling	1.37 ± 0.37	4.62 ± 3.56 ^bdfg^	0.07 ± 0.05	10.9 ± 14.4 ^de^	0.14 ± 0.18	15.5 ± 14.5 ^a^	0.21 ± 0.19
Grilling	1.29 ± 0.22	8.86 ± 6.43 ^eg^	0.11 ± 0.07	7.65 ± 11.3 ^ce^	0.10 ± 0.14	16.5 ± 12.6	0.20 ± 0.15
**Cooking techniques**	**Co**	**CoBA-SI**		**CoBA-LI**		**Total CoBA**	
	**(µg/kg)**	**(%) ^#†^**	**(µg/kg)**	**(%) ^#†^**	**(µg/kg)**	**(%) ^#†^**	**(µg/kg)**
***Plant foods***							
Frying	12.1 ± 12.5	31.2 ± 21.3 ^ABCD^	0.83 ± 0.78	7.13 ± 6.85 ^AB^	0.61 ± 0.51	38.3 ± 23.9 ^ABCD^	4.18 ± 4.82
Raw form	11.2 ± 12.3	27.0 ± 22.5 ^AEFGH^	0.53 ± 0.78	5.27 ± 6.34 ^ACDEF^	0.40 ± 0.43	32.2 ± 23.4 ^AEFGH^	5.25 ± 12.4
Roasting	9.35 ± 7.31	38.5 ± 23.5 ^BEIJ^	0.83 ± 1.14	7.46 ± 6.71 ^CGH^	0.53 ± 0.44	45.9 ± 22.5 ^BEIJ^	5.17 ± 6.44
Toasting	12.3 ± 3.32	11.5 ± 2.27 ^CFIKL^	1.35 ± 0.29	5.23 ± 8.55 ^FGI^	0.67 ± 1.34	16.8 ± 8.65 ^CFIKL^	2.02 ± 1.36
Boiling	7.69 ± 4.23	30.5 ± 16.9 ^GJKM^	0.43 ± 0.28	6.89 ± 6.77 ^DJ^	0.38 ± 0.35	37.4 ± 17.9 ^GJKM^	2.83 ± 2.57
Grilling	6.16 ± 2.62	33.4 ± 18.4 ^DHLM^	0.53 ± 0.50	8.91 ± 6.65 ^BEHIJ^	0.48 ± 0.33	42.3 ± 19.9 ^DHLM^	2.48 ± 1.40
***Animal foods***							
Frying	4.55 ± 2.38	30.2 ± 10.0 ^abc^	1.46 ± 1.18	13.0 ± 11.3 ^abc^	0.45 ± 0.29	43.2 ± 13.9 ^abc^	1.91 ± 1.14
Raw form	3.82 ± 1.32	15.2 ± 9.64 ^adef^	0.69 ± 0.58	11.2 ± 13.6 ^d^	0.33 ± 0.32	26.4 ± 15.4 ^adef^	1.02 ± 0.66
Roasting	4.95 ± 2.17	36.8 ± 25.8 ^dgh^	2.04 ± 2.18	9.99 ± 13.5 ^ae^	0.31 ± 0.23	46.8 ± 29.4 ^dg^	2.36 ± 2.15
Boiling	2.81 ± 1.05	22.3 ± 17.3 ^beg^	0.68 ± 0.59	16.5 ± 13.2 ^bdef^	0.38 ± 0.23	38.9 ± 20.4 ^be^	1.06 ± 0.60
Grilling	3.71 ± 2.17	27.5 ± 27.8 ^cfh^	0.89 ± 0.72	9.61 ± 9.39 ^cf^	0.26 ± 0.20	37.0 ± 26.9 ^cfg^	1.15 ± 0.65
**Cooking techniques**	**Ni**	**NiBA-SI**		**NiBA-LI**		**Total Ni BA**	
	**(µg/kg)**	**(%) ^#†^**	**(µg/kg)**	**(%) ^#†^**	**(µg/kg)**	**(%) ^#†^**	**(µg/kg)**
***Plant foods***							
Frying	196 ± 367	51.1 ± 27.5 ^ABCD^	61.3 ± 11.5	5.12 ± 5.61 ^ABC^	4.35 ± 4.41	56.2 ± 28.6 ^ABCDE^	65.7 ± 11.6
Raw form	132 ± 207	43.7 ± 28.5 ^AEFG^	55.8 ± 12.1	4.95 ± 7.36 ^ADEFG^	2.57 ± 3.10	48.7 ± 30.3 ^AFGH^	58.4 ± 12.1
Roasting	178 ± 315	54.8 ± 27.7 ^EHIJ^	124 ± 29.2	6.27 ± 8.94 ^DH^	5.16 ± 10.1	61.1 ± 26.8 ^BFIJK^	127 ± 29.0
Toasting	196 ± 207	14.5 ± 2.11 ^BFHKL^	28.3 ± 4.11	1.41 ± 0.99 ^BGHIJ^	2.75 ± 1.94	15.9 ± 2.25 ^CGILM^	31.1 ± 4.10
Boiling	102 ± 121	36.0 ± 21.7 ^CGIKM^	39.7 ± 68.3	6.39 ± 8.50 ^EI^	2.76 ± 2.79	42.4 ± 22.0 ^DJLN^	42.4 ± 67.5
Grilling	139 ± 271	45.6 ± 24.4 ^DJLM^	32.5 ± 34.8	6.68 ± 7.05 ^CFJ^	3.34 ± 3.20	52.3 ± 26.6 ^EHKMN^	35.8 ± 34.7
***Animal foods***							
Frying	44.7 ± 18.7	33.9 ± 16.4 ^ab^	15.2 ± 11.7	8.24 ± 8.26 ^ab^	2.95 ± 2.38	42.2 ± 19.9 ^ab^	18.1 ± 11.7
Raw form	48.4 ± 35.0	18.5 ± 11.4 ^acde^	10.4 ± 8.03	9.96 ± 16.9	2.03 ± 2.47	28.5 ± 12.6 ^acd^	12.4 ± 8.77
Roasting	55.3 ± 37.8	39.6 ± 23.4 ^cf^	18.2 ± 18.8	7.25 ± 10.7 ^ade^	1.92 ± 1.92	46.9 ± 28.1 ^ce^	20.1 ± 16.5
Boiling	36.4 ± 13.2	28.9 ± 22.3 ^bdfg^	9.80 ± 7.84	8.64 ± 9.18 ^cdf^	2.73 ± 3.06	37.5 ± 24.6 ^bef^	12.5 ± 8.24
Grilling	110 ± 179	46.7 ± 34.3 ^eg^	84.4 ± 18.3	4.07 ± 5.29 ^bef^	1.79 ± 2.07	50.8 ± 33.6 ^df^	86.0 ± 18.1

^#^ Values are presented as mean ± SD (fresh weight basis). Identical uppercase superscript letters in the columns for the bioaccessibility values of each trace metal analyzed indicate statistically significant differences among different cooking culinary techniques in plant-based foods (*p* < 0.05). ^†^ Values are presented as mean ± SD (fresh weight basis). Identical lowercase superscript letters in the columns for the bioaccessibility values of each trace metal analyzed indicate statistically significant differences among different cooking culinary techniques in animal-based foods (*p* < 0.05).

**Table 3:** Mean large-intestinal bioaccessibility (expressed as solubilized fraction available for absorption, %) of Mn, Co, and Ni (MnBA-LI, CoBA-LI, and NiBA-LI, respectively) in plant- and animal-based foods using fecal inoculum from healthy adults (HE-AD), healthy children (HE-CH), and children with gluten-related disorders (GRD-CH), obesity (OB-CH), and allergy/intolerance to cow’s milk protein (AICM-CH).

Food Group	MnBA-LI in HE-AD	MnBA-LI in HE-CH	Mn-BA-LI in GRD-CH	MnBA-LI in OB-CH	MnBA-LI in AICM-CH
***Plant foods***					
Nuts ^†^	5.25 ± 7.37	5.63 ± 3.48 ^ab^	24.6 ± 8.16 ^acd^	4.34 ± 2.56 ^ce^	30.3 ± 11.8 ^bde^
Cereals ^#†^	3.25 ± 5.12 ^A^	4.00 ± 4.27 ^Aabc^	12.1 ± 10.8 ^ade^	2.29 ± 2.86 ^bdf^	17.6 ± 15.1 ^cef^
Fruits ^#†^	4.34 ± 5.95 ^A^	5.19 ± 3.35 ^Aabc^	15.4 ± 8.38 ^ade^	3.18 ± 2.17 ^bdf^	27.9 ± 19.0 ^cef^
Vegetables ^#†^	4.27 ± 5.40 ^A^	5.17 ± 3.65 ^Aabc^	16.2 ± 10.6 ^ade^	2.79 ± 2.56 ^bdf^	26.2 ± 19.6 ^cef^
Legumes ^#†^	3.82 ± 4.61 ^A^	6.31 ± 3.30 ^Aab^	20.4 ± 12.9 ^ac^	4.10 ± 2.80 ^cd^	30.7 ± 20.4 ^bd^
Oils ^†^	0.20 ± 0.29	0.17 ± 0.15 ^ab^	0.66 ± 0.48 ^ac^	0.07 ± 0.04 ^cd^	1.66 ± 1.55 ^bd^
Beverages + infusions ^†^	0.12 ± 0.15	0.08 ± 0.04 ^abc^	0.33 ± 0.21 ^ad^	0.03 ± 0.04 ^bde^	0.61 ± 0.54 ^ce^
Others ^†^	4.73 ± 3.61	4.40 ± 1.42 ^ab^	16.9 ± 4.89 ^ac^	3.15 ± 1.36 ^cd^	19.2 ± 12.6 ^bd^
***Animal foods***					
Dairy products ^†^	8.15 ± 13.4	9.57 ± 12.5 ^a^	21.5 ± 24.2 ^ab^	7.56 ± 11.5 ^b^	25.6 ± 25.5
Chicken ^†^	4.12 ± 5.15	4.76 ± 3.08 ^ab^	9.98 ± 10.2 ^c^	2.24 ± 1.18 ^ad^	28.1 ± 20.0 ^bcd^
Beef ^†^	3.43 ± 3.35	4.12 ± 2.51 ^ab^	11.1 ± 10.1 ^ac^	2.19 ± 1.59 ^cde^	29.7 ± 19.3 ^bde^
Salmon ^†^	2.84 ± 3.39	2.87 ± 2.85 ^a^	7.09 ± 8.18 ^bc^	1.80 ± 2.45 ^bd^	19.9 ± 17.6 ^acd^
Cod ^†^	2.04 ± 1.62	2.57 ± 2.44 ^ab^	6.09 ± 7.07 ^c^	0.67 ± 0.66 ^acd^	18.0 ± 14.1 ^bd^
Egg ^†^	3.00 ± 2.75	2.40 ± 2.36 ^ab^	6.35 ± 5.94 ^c^	0.46 ± 0.38 ^acd^	16.0 ± 13.4 ^bd^
Lamb ^†^	13.2 ± 2.75	10.4 ± 8.56 ^a^	32.8 ± 18.6 ^ab^	7.17 ± 7.47 ^b^	39.0 ± 31.0
Pork ^†^	5.55 ± 5.64	5.96 ± 3.04 ^ab^	17.0 ± 13.6 ^ac^	2.78 ± 1.57 ^bcd^	25.4 ± 20.1 ^d^
**Food group**	**CoBA-LI in HE-AD**	**CoBA-LI in** **HE-CH**	**Co-BA-LI in** **GRD-CH**	**CoBA-LI in** **OB-CH**	**CoBA-LI in** **AICM-CH**
***Plant foods***					
Nuts ^#†^	4.38 ± 4.99 ^A^	9.73 ± 5.04 ^Aab^	5.75 ± 5.26 ^a^	5.02 ± 4.30 ^b^	6.66 ± 4.35
Cereals ^#†^	2.64 ± 4.41 ^A^	4.71 ± 3.93 ^Aab^	2.94 ± 3.50 ^a^	2.77 ± 3.21 ^b^	4.31 ± 7.53
Fruits ^#†^	8.28 ± 7.83 ^A^	14.0 ± 8.85 ^Aabc^	9.06 ± 6.31 ^ad^	7.66 ± 7.29 ^bde^	10.4 ± 6.67 ^ce^
Vegetables ^#†^	5.99 ± 6.62 ^A^	12.4 ± 7.21 ^Aabc^	6.63 ± 5.06 ^ad^	6.21 ± 5.46 ^be^	7.94 ± 5.42 ^cde^
Legumes ^#†^	4.54 ± 4.92 ^A^	10.4 ± 7.10 ^Aa^	7.36 ± 7.02	4.99 ± 5.38 ^a^	7.01 ± 4.47
Oils ^#†^	0.13 ± 0.18 ^A^	0.26 ± 0.22 ^Aa^	0.15 ± 0.14	0.11 ± 0.11 ^a^	0.16 ± 0.12
Beverages + infusions ^#†^	0.08 ± 0.11 ^A^	0.11 ± 0.05 ^Aabc^	0.06 ± 0.06 ^a^	0.05 ± 0.05 ^b^	0.05 ± 0.03 ^c^
Others ^#^	1.20 ± 0.74 ^A^	2.36 ± 0.72 ^A^	1.74 ± 0.91	1.43 ± 0.98	1.73 ± 0.67
***Animal foods***					
Dairy products	9.05 ± 8.94	16.3 ± 13.5	10.8 ± 12.2	10.7 ± 13.9	11.3 ± 10.5
Chicken ^†^	10.8 ± 14.1	20.1 ± 15.4 ^a^	15.0 ± 13.2	8.59 ± 6.94 ^a^	15.0 ± 13.9
Beef	8.53 ± 5.40	13.0 ± 8.10	8.85 ± 7.04	9.09 ± 8.39	10.8 ± 5.51
Salmon	5.24 ± 5.35	8.46 ± 7.17	5.04 ± 4.88	5.59 ± 6.94	5.67 ± 4.54
Cod ^†^	5.80 ± 3.58	8.22 ± 4.34 ^a^	5.03 ± 3.47	3.89 ± 4.16 ^ab^	6.52 ± 2.37 ^b^
Egg	9.33 ± 6.37	12.4 ± 9.15	5.63 ± 5.27	6.29 ± 6.76	7.62 ± 5.63
Lamb	24.5 ± 15.8	32.9 ± 17.1	25.0 ± 16.3	26.9 ± 16.8	24.5 ± 14.3
Pork ^#^	11.3 ± 12.0 ^A^	23.6 ± 17.9 ^A^	14.2 ± 14.1	15.4 ± 15.0	13.7 ± 13.0
**Food group**	**NiBA-Li in** **HE-AD**	**NiBA-LI in** **HE-CH**	**NiBA-LI in** **GRD-CH**	**NiBA-LI in** **OB-CH**	**NiBA-LI in** **AICM-CH**
***Plant foods***					
Nuts ^†^	2.18 ± 1.86	1.82 ± 1.22 ^a^	1.85 ± 2.00	0.94 ± 0.72 ^ab^	2.06 ± 2.03 ^b^
Cereals ^†^	2.56 ± 4.75	2.78 ± 4.31 ^a^	2.73 ± 4.10 ^b^	1.36 ± 2.34 ^abc^	2.30 ± 3.75 ^c^
Fruits ^#†^	11.0 ± 9.82 ^A^	7.53 ± 7.87 ^Aa^	6.52 ± 6.63 ^b^	3.64 ± 4.24 ^abc^	8.20 ± 7.52 ^c^
Vegetables ^#†^	12.8 ± 11.9 ^A^	7.21 ± 6.72 ^Aa^	6.12 ± 5.84 ^bc^	3.84 ± 7.11 ^abd^	7.84 ± 5.98 ^cd^
Legumes	2.96 ± 2.33	2.56 ± 2.46	3.89 ± 5.48	1.43 ± 1.58	2.98 ± 3.59
Oils ^†^	0.10 ± 0.10	0.09 ± 0.08 ^a^	0.09 ± 0.09 ^b^	0.02 ± 0.03 ^abc^	0.09 ± 0.10 ^c^
Beverages + infusions ^†^	0.13 ± 0.30	0.08 ± 0.08 ^a^	0.09 ± 0.10 ^b^	0.02 ± 0.03 ^ab^	0.06 ± 0.09
Others	1.08 ± 1.04	1.33 ± 1.01	1.33 ± 0.93	0.61 ± 0.73	1.17 ± 0.93
***Animal foods***					
Dairy products ^†^	10.4 ± 13.8	9.49 ± 11.5 ^a^	10.5 ± 16.0	6.23 ± 15.1 ^a^	8.44 ± 14.2
Chicken ^#†^	9.23 ± 4.06 ^A^	5.36 ± 2.94 ^Aa^	7.58 ± 7.39 ^b^	2.25 ± 2.18 ^ab^	5.90 ± 6.08
Beef ^#^	16.9 ± 14.2 ^A^	5.34 ± 6.78 ^A^	7.96 ± 8.87	4.30 ± 4.78	7.93 ± 10.0
Salmon ^#^	13.4 ± 12.8 ^A^	5.92 ± 8.44 ^A^	7.88 ± 10.1	4.64 ± 8.35	5.91 ± 9.52
Cod ^#^	7.50 ± 3.02 ^A^	2.01 ± 1.75 ^A^	2.69 ± 2.40	1.50 ± 1.81	2.98 ± 3.12
Egg	10.4 ± 12.5	3.19 ± 3.12	2.50 ± 2.77	1.22 ± 1.65	3.22 ± 4.06
Lamb ^†^	10.9 ± 13.4	14.3 ± 12.9 ^a^	13.4 ± 14.0 ^b^	4.96 ± 6.01 ^ab^	10.8 ± 13.2
Pork ^†^	8.18 ± 10.5	11.6 ± 6.38 ^a^	9.68 ± 8.57	5.24 ± 6.87 ^a^	7.36 ± 8.52

^#^ Values are presented as mean ± SD. Rows with identical uppercase superscript letters for bioaccessibility values of each trace metal indicate statistically significant differences between HE-AD and HE-CH among each food category in plant- and animal-based foods (*p* < 0.0001). ^†^ Values are presented as mean ± SD. Rows with identical lowercase superscript letters for bioaccessibility values of each trace metal indicate statistically significant differences among different children’s groups for each food category in plant- and animal-based foods (*p* < 0.05).

**Table 4:** Serving size, Mn, Co, and Ni concentrations (expressed as the measured concentration of the element in the soluble fraction, mg/kg or µg/kg), and intakes per serving (serv.), and percentage coverage of the dietary reference intakes (DRIs) for Mn, the provisional chronic oral reference dose (RfD) for Co, and the tolerable upper intake levels (TUILs) for Ni in different foodstuffs.

Food Group	Serving Weight	Mn		Mn Estimated Intake/Serving		Co		Co Estimated Intake/Serving		Ni		Ni Estimated Intake/Serving	
				Men/Women	Children 4–8 y			Adults	Children 4–8 y			Adults	Children 4–8 y
	(g)	(mg/kg)	(mg/serv.)	(% RDI) ^#^		(µg/kg)	(µg/serv.)	(% RfD) ^†^		(µg/kg)	(µg/serv.)	(% TUIL) *	
***Vegetables and fruits***													
Zucchini	175	2.20 ± 0.27	0.38	16.7/21.4	25.6	6.07 ± 1.81	1.06	5.06	19.7–13.1	157 ± 61.6	27.6	2.75	9.18
Capsicum	175	1.88 ± 0.55	0.33	14.3/18.3	21.9	7.05 ± 2.60	1.23	5.87	22.8–15.2	46.3 ± 22.0	8.10	0.81	2.70
Carrot	175	1.36 ± 0.45	0.24	10.3/13.2	15.9	5.55 ± 1.79	0.97	4.62	18.0–12.0	69.4 ± 33.4	12.1	1.21	4.05
Potatoes	175	2.10 ± 0.62	0.37	16.0/20.4	24.5	5.91 ± 1.57	1.03	4.92	19.2–12.8	73.3 ± 37.9	12.9	1.28	4.28
Sweet potatoes	175	1.96 ± 0.33	0.34	14.9/19.1	22.9	3.80 ± 0.42	0.66	3.17	12.3–8.21	41.4 ± 3.87	7.24	0.72	2.41
Eggplant	175	2.64 ± 0.66	0.46	20.1/25.7	30.8	9.87 ± 3.14	1.73	8.22	32.0–21.3	91.2 ± 28.2	16.0	1.60	5.32
Onion	175	2.57 ± 0.62	0.45	19.6/25.0	29.9	9.73 ± 3.75	1.70	8.11	31.5–21.0	45.2 ± 22.4	7.91	0.79	2.64
Cauliflower	175	2.42 ± 0.76	0.42	18.4/23.5	28.2	13.6 ± 3.66	2.38	11.3	44.1–29.4	57.4 ± 30.5	10.0	1.00	3.34
Spinach	175	3.64 ± 1.88	0.64	27.7/35.4	42.5	9.74 ± 6.64	1.70	8.12	31.6–21.0	452 ± 459	79.1	7.91	26.4
Garlic	5	2.87 ± 0.50	0.01	0.61/0.78	0.93	8.84 ± 0.90	0.04	0.21	0.81–0.54	54.7 ± 15.3	0.27	0.03	0.09
Tomatoes	175	1.95 ± 0.81	0.34	14.8/19.0	22.7	6.75 ± 3.19	1.18	5.62	21.9–14.6	50.5 ± 25.5	8.84	0.88	2.95
Cabbage	175	1.40 ± 0.26	0.24	10.7/13.6	16.3	4.35 ± 1.58	0.76	3.62	14.1–9.40	32.5 ± 9.23	5.69	0.57	1.90
Lettuce	175	1.02 ± 0.11	0.18	7.76/9.91	11.9	5.67 ± 0.37	0.99	4.72	18.4–12.2	14.4 ± 2.36	2.52	0.25	0.84
Apple	160	2.37 ± 0.51	0.38	16.4/21.1	25.3	6.85 ± 2.56	1.10	5.22	20.3–13.5	221 ± 65.3	35.4	3.54	11.8
Banana	160	2.03 ± 0.38	0.32	14.1/18.0	21.7	4.50 ± 1.35	0.72	3.43	13.3–8.89	46.4 ± 7.78	7.42	0.74	2.47
Orange	160	1.50 ± 0.21	0.24	10.4/13.3	16.0	6.21 ± 2.14	0.99	4.73	18.4–12.3	42.7 ± 35.5	6.83	0.68	2.28
Grapes	160	1.20 ± 0.19	0.19	8.35/10.7	12.8	6.39 ± 6.63	1.02	4.87	18.9–12.6	36.0 ± 20.3	5.76	0.57	1.92
Plum	160	1.89 ± 0.35	0.30	13.1/16.8	20.2	5.36 ± 1.81	0.86	4.08	15.9–10.6	19.8 ± 7.52	3.17	0.32	1.06
Peach	160	1.59 ± 0.18	0.25	11.1/14.1	17.0	4.74 ± 1.09	0.76	3.61	14.0–9.36	46.1 ± 15.3	7.38	0.73	2.46
Olives	30	1.80 ± 0.17	0.07	3.13/4.00	4.80	8.07 ± 1.15	0.32	1.54	5.98–3.99	43.5 ± 2.33	1.74	0.17	0.58
***Cereals and by-products***													
Bread	50	3.51 ± 1.32	0.18	7.63/9.75	11.7	28.3 ± 24.2	1.41	6.74	26.2–17.5	716 ± 372	35.8	3.58	11.9
Whole wheat bread	50	4.98 ± 0.99	0.25	10.8/13.8	16.6	17.3 ± 2.29	0.86	4.12	16.0–10.7	237 ± 44.9	11.9	1.18	3.95
Penne	70	1.45 ± 0.20	0.10	4.41/5.64	6.77	9.89 ± 0.38	0.69	3.30	12.8–8.55	111 ± 2.29	7.77	0.78	2.59
Whole grain penne	70	2.86 ± 1.07	0.20	8.70/11.1	13.3	10.4 ± 2.77	0.728	3.47	13.5–8.99	164 ± 97.4	11.5	1.15	3.83
Rice	70	3.02 ± 0.19	0.21	9.19/11.7	14.1	20.7 ± 0.10	1.45	6.90	26.8–17.9	556 ± 0.47	38.9	3.89	13.0
Whole grain rice	70	3.08 ± 0.98	0.22	9.38/12.0	14.4	10.9 ± 0.13	0.77	3.63	14.1–9.42	156 ± 1.78	10.9	1.09	3.64
Cookies	70	1.63 ± 0.60	0.11	4.96/6.34	7.61	12.4 ± 2.04	0.87	4.13	16.1–10.7	31.2 ± 3.21	2.18	0.22	0.73
Whole grain cookies	70	2.80 ± 0.24	0.20	8.52/10.9	13.1	8.81 ± 0.76	0.62	2.94	11.4–7.61	43.0 ± 1.51	3.01	0.30	1.00
Whole grain breakfast cereals	70	0.99 ± 0.50	0.07	3.01/3.85	4.62	8.67 ± 1.53	0.61	2.89	11.2–7.49	54.1 ± 11.6	3.79	0.38	1.26
Breakfast cereals	70	2.99 ± 0.75	0.21	9.1/11.6	14.0	9.79 ± 1.23	0.69	3.26	12.7–8.46	62.0 ± 2.55	4.34	0.43	1.45
***Animal-based foods***													
Butter	10	0.76 ± 0.29	0.01	0.33/0.42	0.51	2.83 ± 0.75	0.03	0.14	0.52–0.35	39.8 ± 2.04	0.40	0.04	0.13
Gouda cheese	50	1.37 ± 0.23	0.07	2.98/3.81	4.57	5.50 ± 2.87	0.27	1.31	5.09–3.40	69.1 ± 23.6	3.45	0.35	1.15
Milk	225	1.23 ± 0.04	0.28	12.0/15.4	18.4	3.77 ± 0.01	0.85	4.04	15.8–10.5	24.5 ± 0.07	5.51	0.55	1.84
Yogurt	225	1.78 ± 2.66	0.40	17.3/22.1	26.5	2.69 ± 1.33	0.60	2.88	11.2–7.47	2.55 ± 1.89	0.57	0.06	0.19
Chicken	113	1.21 ± 0.23	0.14	5.91/7.56	9.08	2.63 ± 1.20	0.30	1.41	5.48–3.65	36.8 ± 8.29	4.14	0.41	1.38
Beef	113	1.50 ± 0.23	0.17	7.34/9.38	11.2	4.03 ± 1.00	0.45	2.16	8.40–5.60	167 ± 242	18.8	1.88	2.16
Salmon	138	1.46 ± 0.33	0.20	8.73/11.2	13.4	5.75 ± 1.74	0.79	3.76	14.6–9.76	55.0 ± 32.5	7.56	0.76	3.16
Cod	138	1.68 ± 0.39	0.23	10.0/12.8	15.4	4.26 ± 1.29	0.59	2.79	10.8–7.23	56.9 ± 8.96	7.82	0.78	2.61
Egg	58	1.66 ± 0.50	0.10	4.19/5.35	6.42	4.20 ± 2.34	0.24	1.16	4.51–3.01	65.8 ± 35.7	3.82	0.38	1.27
Lamb	113	0.90 ± 0.21	0.10	4.40/5.62	6.75	2.31 ± 0.83	0.26	1.24	4.81–3.21	34.1 ± 15.4	3.84	0.38	1.28
Pork	113	1.18 ± 0.31	0.13	5.77/7.37	8.85	4.11 ± 2.54	0.46	2.20	8.56–5.71	29.9 ± 10.5	3.36	0.34	1.12
***Other foods (mixed nuts, legumes, oils, chocolate, Nutella, wine, beer, cola, and coffee)***													
Nuts mix	25	5.42 ± 1.03	0.14	5.89/7.53	9.03	26.1 ± 6.11	0.65	3.11	12.1–8.06	833 ± 97.8	20.8	2.08	6.94
Peanuts	25	2.27 ± 0.30	0.06	2.47/3.15	3.78	8.22 ± 1.76	0.20	0.98	3.81–2.54	185 ± 46.1	4.62	0.46	1.54
Beans	70	1.64 ± 0.12	0.11	4.99/6.38	7.65	5.69 ± 0.82	0.40	1.90	7.38–4.92	87.7 ± 19.0	6.14	0.61	2.05
Lentils	70	2.01 ± 0.47	0.14	6.12/7.82	9.38	15.8 ± 10.1	1.11	5.27	20.5–13.7	566 ± 592	39.6	3.96	13.2
Olive oil	10	0.45 ± 0.15	0.01	0.20/0.25	0.30	4.46 ± 1.30	0.04	0.21	0.83–0.55	68.1 ± 24.9	0.68	0.07	0.23
Sunflower oil	10	0.45 ± 0.41	0.01	0.20/0.25	0.30	3.72 ± 2.68	0.04	0.18	0.69–0.46	34.2 ± 0.61	0.34	0.03	0.11
Dark chocolate	20	4.52 ± 0.31	0.09	3.93/5.02	6.03	68.7 ± 0.52	1.37	6.54	25.4–17.0	544 ± 4.61	10.9	1.09	3.63
Nutella	20	2.14 ± 0.42	0.04	1.86/2.38	2.85	44.3 ± 0.32	0.89	4.22	16.4–10.9	684 ± 6.81	13.7	1.37	4.56
Red wine	100	0.44 ± 0.02	0.04	1.91/2.44	2.93	2.80 ± 0.03	0.28	1.33	5.19–3.46	72.7 ± 0.42	7.27	0.73	2.42
Beer	200	1.27 ± 1.54	0.25	11.0/14.1	16.9	12.0 ± 0.07	2.40	11.4	44.4–29.6	96.3 ± 0.23	19.3	1.93	6.42
Coke	333	0.87 ± 1.27	0.29	12.6/16.1	19.3	7.38 ± 0.03	0.25	11.7	45.5–30.3	16.1 ± 0.06	5.36	0.54	1.79
Light coke	333	1.52 ± 0.47	0.51	22.0/28.1	33.7	6.93 ± 0.03	2.31	11.0	42.7–28.5	86.9 ± 0.06	28.9	2.89	9.64
Coffee	100	0.85 ± 0.02	0.08	3.65/4.67	5.60	10.0 ± 0.01	1.00	4.76	18.5–12.3	41.3 ± 0.03	4.13	0.41	1.38
Instant coffee	100	1.16 ± 0.02	0.12	5.04/6.44	7.73	15.3 ± 0.05	1.53	7.29	28.3–18.9	64.7 ± 0.53	6.47	0.65	2.16

^#^ Mn RDI established by the IOM [59] for adult men, adult women, and children aged 3–8 years (2.3, 1.8, and 1.5 mg/day, respectively). ^†^ RfD for Co: 0.0003 mg/kg/day; equivalent to ~21 µg/day for a 70 kg adult and 5.4–8.1 µg/day for children aged 4–8 years (18–27 kg). * TUILs for Ni in humans: 1000 µg/day for adults and 300 µg/day for children aged 4–8 years [4].

## Data Availability

The raw data supporting the conclusions of this article will be made available by the authors on request.

## Supplementary Materials

The following supporting information can be downloaded at https://www.mdpi.com/article/10.3390/foods15203599/s1: Table S1: Food samples considered in the study, either in their raw form or after being submitted to different culinary processes (*n*: number of samples) [30]; Table S2: Concentration (mM/L) and composition of the salts in the stock solutions, and their respective concentrations (mM/L) in the simulated oral, gastric and intestinal fluids used in the in vitro model [30]; Table S3: Analytical performance parameters, including the limits of detection (LOD) and quantification (LOQ), sensitivity, precision (expressed as repeatability), and accuracy for the analyzed elements determined by ICP-MS, as assessed using the certified reference materials (CRMs) NIST 1515 Apple Leaves, ERM-CE278k Mussel Tissue, and BCR-679 White Cabbage; Table S4: Mean Mn, Co, and Ni concentrations (mg/kg for Mn and µg/kg for Co and Ni; fresh weight; mean ± standard deviation, SD), partial bioaccessibility values (MnBA, CoBA, and NiBA; %, mean ± SD) in the small and large intestine (MnBA-SI/MnBA-LI, CoBA-SI/CoBA-LI, and NiBA-SI/NiBA-LI, respectively), and total MnBA, CoBA, and NiBA in plant-based foods categorized according to their protein content (high-protein: legumes, nuts, and cereals; low-protein: vegetables and fruits); Table S5: Mean Mn, Co, and Ni concentrations (mg/kg for Mn and µg/kg for Co and Ni; fresh weight; mean ± standard deviation, SD), partial bioaccessibility values (MnBA, CoBA, and NiBA; %, mean ± SD) in the small and large intestine (MnBA-SI/MnBA-LI, CoBA-SI/CoBA-LI, and NiBA-SI/NiBA-LI, respectively), and total MnBA, CoBA, and NiBA in whole-grain and refined cereal products; Table S6: Mean Mn, Co, and Ni concentrations (mg/kg for Mn and µg/kg for Co and Ni; fresh weight; mean ± standard deviation, SD), partial bioaccessibility values (MnBA, CoBA, and NiBA; %, mean ± SD) in the small and large intestine (MnBA-SI/MnBA-LI, CoBA-SI/CoBA-LI, and NiBA-SI/NiBA-LI, respectively), and total MnBA, CoBA, and NiBA in gluten-containing and gluten-free cereals; Table S7: Mean concentrations of Mn (mg/kg, fresh weight), Co, and Ni (µg/kg, fresh weight); partial bioaccessibility values in the small intestine (MnBA-SI, CoBA-SI, and NiBA-SI) and large intestine (MnBA-LI, CoBA-LI, and NiBA-LI); and total bioaccessibility (MnBA, CoBA, and NiBA) in vegetables and fruits (mean ± standard deviation, SD; %); Table S8: Mean and range of Mn (mg/kg) and Co and Ni (µg/kg) concentrations, corresponding bioaccessibility values (MnBA, CoBA, and NiBA, respectively; %) reported in foods, as well as daily dietary intakes and risk assessments established for these elements in other studies. Figure S1. Box-and-Whisker plots for total and partial bioaccessibility values (%; total BA; BA in the small intestine, and in the large intestine: BA-SI, and BA-LI; respectively) of the trace metals analyzed in plant- and animal-based foods: (S1a) Mn bioaccessibility in the small, and in the large intestine (MnBA-SI, and MnBA-LI; respectively); (S1b) Total Mn bioaccessibility (Total MnBA); (S1c) CoBA-SI, and CoBA-LI; (S1d) Total CoBA; (S1e) NiBA-SI, and NiBA-LI; (S1f) Total NiBA; Figure S2: Box-and-Whisker plots for partial bioaccessibility values (BA in the small intestine, and in the large intestine: BA-SI, and BA-LI; respectively) of the trace metals analyzed in plant-based foods according to grouped cooking techniques (frying + boiling, raw form, and roasting + grilling): (S2a) Mn bioaccessibility in the small, and in the large intestine (MnBA-SI and MnBA-LI, respectively); (S2b) CoBA-SI and CoBA-LI; (S2c) NiBA-SI and NiBA-LI; Figure S3. Box-and-Whisker plots for partial bioaccessibility values (BA in the small intestine, and in the large intestine: BA-SI, and BA-LI; respectively) of the trace metals analyzed in animal-based foods according to grouped cooking techniques (frying + boiling, raw form, and roasting + grilling): (S3a) Mn bioaccessibility in the small, and in the large intestine (MnBA-SI and MnBA-LI, respectively); (S3b) CoBA-SI and CoBA-LI; (S3c) NiBA-SI and NiBA-LI; Figure S4. Box-and-Whisker plots for partial bioaccessibility values (BA in the small intestine, and in the large intestine: BA-SI, and BA-LI; respectively) of the trace metals analyzed in vegetables according to cooking technique: (S4a) Mn bioaccessibility in the small, and in the large intestine (MnBA-SI and MnBA-LI, respectively); (S4b) CoBA-SI and CoBA-LI; (S4c) NiBA-SI and NiBA-LI; Figure S5. Box-and-Whisker plots for bioaccessibility values in the large intestine (BA-LI) of the trace metals analyzed in all foodstuffs using fecal inoculum from healthy adults (HE-AD), healthy children (HE-CH), children with gluten-related disorders (GRD-CH), children with obesity (OB-CH), and children with allergy/intolerance to cow’s milk proteins (AICM-CH): (S5a) Mn bioaccessibility in the small, and in the large intestine (MnBA-SI and MnBA-LI, respectively); (S5b) CoBA-SI and CoBA-LI; (S5c) NiBA-SI and NiBA-LI.

## Abbreviations

The following abbreviations are used in this manuscript:

MnBA	Bioaccessibility of Mn
CoBA	Bioaccessibility of Co
NiBA	Bioaccessibility of Ni
HE-AD	Healthy adults
HE-CH	Healthy children
GRD-CH	Children with gluten-related disorders
OB-CH	Children with obesity
AICM-CH	Children with allergy or intolerance to cow’s milk proteins
MnBA-SI	Bioaccessibility of Mn in the small intestine
CoBA-SI	Bioaccessibility of Co in the small intestine
NiBA-SI	Bioaccessibility of Ni in the small intestine
MnBA-LI	Bioaccessibility of Mn in the large intestine
CoBA-LI	Bioaccessibility of Co in the large intestine
NiBA-LI	Bioaccessibility of Ni in the large intestine
DRIs	Dietary reference intakes
RfD	Provisional chronic oral reference dose
TUIL	Tolerable Upper Intake Levels
