# Supplementary material for: Bioaccessibility via In Vitro Digestion and Colonic Fermentation, Intake, and per Serving Risk Assessment of Mn, Co, and Ni in Cooked Food
Source: Foods. 2026 Oct 10;15(20):3599. doi: 10.3390/foods15203599 (PMC13703824; doi:10.3390/foods15203599)
Supplement: Supplementary file 1 [file foods-15-03599-s001.zip › foods-4585409-supplementary.pdf]

Bioaccessibility via In Vitro Digestion and Colonic Fermentation, Intake, and per Serving Risk Assessment of Mn, Co, and Ni in Cooked Food

Miguel Navarro-Moreno, José Ángel Rufián-Henares, Konstantinos Douros, Francisco M. Ocaña-Peinado, Miguel Navarro-Alarcón and Silvia Pastoriza

Supplementary Materials

Table S1. Food samples considered in the study, either in their raw form or after being submitted to different culinary processes (*n*: number of samples) [30].

Food type	Food group	Food item	Culinary processes or raw form	n
Animal	Meat	Chicken, beef, lamb, pork	Frying, roasting, boiling, grilling	16
	Fish	Salmon	Frying, roasting, boiling, grilling, raw form	5
		Cod	Frying, roasting, boiling, grilling	4
	Dairy	Butter	Frying, raw form	2
		Milk (cow milk, fermented cow milk)	Raw form	2
		Gouda cheese	Frying, roasting, grilling, raw form	4
	Egg	Egg	Frying, roasting, boiling, grilling	4
Vegetal	Fruits	Apple, banana, orange, grapes, plum, peach	Frying, roasting, grilling, raw form	24
		Olive	Raw form	1
	Cereals	Regular bread, whole-grain bread	Frying, toasting, raw form	6
		Penne, whole-grain penne	Boiling	2
		Rice, whole-grain rice	Boiling	2
		Biscuits, whole-grain biscuits	Raw form	2
		Breakfast cereals, whole-grain breakfast cereals	Raw form	2
	Nuts	Nuts mixture, peanuts	Frying, roasting, raw form	6
	Legumes	Lentils, kidney beans	Roasting, boiling, grilling	6
	Vegetables	Zucchini, pepper, carrot, eggplant, onion, cauliflower, tomato, spinach, garlic, cabbage	Frying, roasting, boiling, grilling, raw form	50

	Lettuce	Raw form	1
	Tubers (potato, sweet potato)	Frying, boiling, grilling	8
Oils	Sunflower oil, olive oil	Frying	4
Beverages and infusions	Beer, red wine, cola (regular cola, light cola), coffee (regular coffee, instant coffee)	Crude form	6
Others	Dark chocolate, hazelnut spread	Raw form	2

Table S2. Concentration (mM/L) and composition of the salts in the stock solutions, and their respective concentrations (mM/L) in the simulated oral, gastric and intestinal fluids used in the in vitro model [30].

**Salt composition**	**Salt concentration in the stock solution**	**Salt concentration in the simulated oral fluid (pH = 7)**	**Salt concentration in the simulated gastric fluid (pH = 3)**	**Salt concentration in the simulated intestinal fluid (pH = 7)**
KCl	0.5	15.09	6.9	6.8
KH_2PO_4	0.5	1.35	0.9	0.8
NH_4CO_3	1	13.68	25	85
NaCl	2	-	47.2	38,4
$MgCl_2.(H_2O)_6$	0.15	0.15	0.12	0.33
$NH_4(CO_3)_2$	0.5	0.06	0.5	-
$CaCl_2.(H_2O)_2$	0.3	1.5	0.15	0.6
HCl	6	1.1	15.6	8.4

Table S3. Analytical performance parameters, including the limits of detection (LOD) and quantification (LOQ), sensitivity, precision (expressed as repeatability), and accuracy for the analyzed elements determined by ICP-MS, as assessed using the certified reference materials (CRMs) NIST 1515 Apple Leaves, ERM-CE278k Mussel Tissue, and BCR-679 White Cabbage.

**Analitycal parameters**	**Mn**	**Co**	**Ni**
LOD (µg/L)	0.032	0.001	0.016
LOQ (µg/L)	0.096	0.003	0.048
Accuracy			
NIST 1515 (mean ± SD, µg/g			
Certified level (mean ± SD, µg/g)	54.1± 1.10	0.090 #	0.936 ± 0.094
Found level (mean ± SD, µg/g)	52.3 ± 4.02	0.096 ± 0.020	0.895 ± 0.077
Recovery (%)	96.7	106.7	95.6
ERM - CE278k			
Certified level (mean ± SD, µg/g)	4.88 ± 0.24	0.210#	0.690 ± 0.15
Found level (mean ± SD, µg/g)	4.75 ± 0.22	0. 202 ± 0.01	0.705 ± 0.07
Recovery (%)	97.3	96.2	102.2
BCR 679			
Certified level (mean ± SD, µg/g)	13.3 ± 0.50	-	27.0 ± 0.80
Found level (mean ± SD, µg/g)	13.6 ± 0.60	-	26.4 ± 0.85
Recovery (%)	102.6	-	97.8
Selectivity	1.02	0.96	0.98
Repeatability (RSD, %)	4.23-7.30	8.27-12.1	7.32-10.4

Non-certified Co level.

Table S4. Mean Mn, Co, and Ni concentrations (mg/kg for Mn and μg/kg for Co and Ni; fresh weight; mean ± standard deviation, SD), partial bioaccessibility values (MnBA, CoBA, and NiBA; %, mean ± SD) in the small and large intestine (MnBA-SI/MnBA-LI, CoBA-SI/CoBA-LI, and NiBA-SI/NiBA-LI, respectively), and total MnBA, CoBA, and NiBA in plant-based foods categorized according to their protein content (high-protein: legumes, nuts, and cereals; low-protein: vegetables and fruits).

**Protein content**	**Mn (mg/kg)**#	**MnBA-SI (%)**#	**MnBA-LI (%)**#	**Total MnBA (%)**#
High protein	3.01 ± 1.53^{A}	35.4 ± 17.9^{B}	10.8 ± 13.5^{C}	46.2 ± 23.3^{D}
Low protein	1.99 ± 0.97^{A}	21.8 ± 15.8^{B}	9.84 ± 13.3^{C}	31.6 ± 21.3^{D}
**Protein content**	**Co (μg/kg)**#	**CoBA-SI (%)**	**CoBA-LI (%)**#	**Total CoBA (%)**
High protein	15.2 ± 11.9^{A}	33.7 ± 24.6	4.91 ± 5.41^{B}	38.6 ± 25.3
Low protein	8.12 ± 8.14^{A}	30.6 ± 20.3	7.43 ± 7.00^{B}	38.0 ± 21.7
**Protein content**	**Ni (μg/kg)**#	**NiBA-SI (%)**	**NiBA-LI (%)**#	**Total NiBA (%)**#
High protein	348 ± 432^{A}	45.9 ± 30.4	2.31 ± 3.42^{B}	48.2 ± 31.0^{C}
Low protein	96.4 ± 163^{A}	45.7 ± 26.3	6.60 ± 8.03^{B}	52.3 ± 27.5^{C}

The presence of identical uppercase superscript letters in the columns for the concentrations and bioaccessibility values of every trace metal analyzed (Mn, Co and Ni) indicate statistically significant differences on the Kruskal-Wallis and Mann-Whitney U test ($p < 0.05$).

Table S5. Mean Mn, Co, and Ni concentrations (mg/kg for Mn and µg/kg for Co and Ni; fresh weight; mean ± standard deviation, SD), partial bioaccessibility values (MnBA, CoBA, and NiBA; %, mean ± SD) in the small and large intestine (MnBA-SI/MnBA-LI, CoBA-SI/CoBA-LI, and NiBA-SI/NiBA-LI, respectively), and total MnBA, CoBA, and NiBA in whole-grain and refined cereal products.

**Cereal group**	**Mn (mg/kg)#**	**MnBA-SI (%)**	**MnBA-LI (%)#**	**Total MnBA (%)**
Whole cereals	3.52 ± 1.56^{A}	31.1 ± 21.9	9.58 ± 12.5^{B}	40.7 ± 33.6
Regular cereals	2.80 ± 1.20^{A}	29.5 ± 12.5	6.12 ± 8.20^{B}	35.7 ± 15.9
**Cereal group**	**Co (µg/kg)**	**CoBA-SI (%)#**	**CoBA-LI (%)#**	**Total CoBA (%)#**
Whole cereals	12.9 ± 4.12	23.3 ± 22.7^{A}	4.61 ± 6.13^{B}	27.9 ± 23.0^{C}
Regular cereals	19.7 ± 17.8	17.4 ± 15.3^{A}	2.34 ± 2.50^{B}	19.8 ± 15.0^{C}
**Cereal group**	**Ni (µg/kg)**	**NiBA-SI (%)**	**NiBA-LI (%)#**	**Total NiBA (%)**
Whole cereals	161 ± 83.9	25.3 ± 28.7	2.73 ± 4.29^{A}	28.1 ± 30.2
Regular cereals	416 ± 578	35.4 ± 29.5	1.96 ± 3.52^{A}	37.3 ± 31.1

The presence of columns labelled with identical superscript uppercase letters for the concentrations and bioaccessibility values of every trace metal analyzed (Mn, Co and Ni) indicate statistically significant differences on the Kruskal-Wallis and Mann-Whitney U test ($p < 0.05$).

Table S6. Mean Mn, Co, and Ni concentrations (mg/kg for Mn and μg/kg for Co and Ni; fresh weight; mean ± standard deviation, SD), partial bioaccessibility values (MnBA, CoBA, and NiBA; %, mean ± SD) in the small and large intestine (MnBA-SI/MnBA-LI, CoBA-SI/CoBA-LI, and NiBA-SI/NiBA-LI, respectively), and total MnBA, CoBA, and NiBA in gluten-containing and gluten-free cereals.

Cereal group	Mn (mg/kg)[#]	MnBA-SI (%)[#]	MnBA-LI (%)[#]	Total MnBA (%)[#]
Gluten cereals	3.18 ± 1.55[A]	32.9 ± 17.9[B]	8.56± 11.2[C]	41.5 ± 19.9[D]
Gluten-free cereals	3.05 ± 0.03[A]	14.6 ± 2.64[B]	3.56 ± 4.70[C]	18.2 ± 4.02[D]
**Cereal group**	**Co (μg/kg)[#]**	**CoBA-SI (%)[#]**	**CoBA-LI (%)[#]**	**Total CoBA (%)[#]**
Gluten cereals	16.4 ± 14.2[A]	13.2 ± 7.75[B]	3.92 ± 5.05[C]	17.1 ± 10.7[D]
Gluten-free cereals	15.8 ± 5.01[A]	63.5 ± 11.3[B]	0.80 ± 0.85[C]	64.3 ± 11.8[D]
**Cereal group**	**Ni (μg/kg)[#]**	**NiBA-SI (%)[#]**	**NiBA-LI (%)[#]**	**Total NiBA (%)[#]**
Gluten cereals	277 ± 458[A]	28.6 ± 30.9[B]	2.68 ± 4.16[C]	31.3 ± 32.7[D]
Gluten-free cereals	356± 203[A]	41.1 ± 14.8[B]	0.30 ± 0.31[C]	41.4 ± 14.5[D]

[#] The presence of columns labelled with identical superscript uppercase letters for the concentrations and bioaccessibility values of every trace metal analyzed (Mn, Co and Ni) indicate statistically significant differences on the Kruskal-Wallis and Mann-Whitney U test ($p < 0.05$).

Table S7. Mean concentrations of Mn (mg/kg, fresh weight), Co, and Ni (µg/kg, fresh weight); partial bioaccessibility values in the small intestine (MnBA-SI, CoBA-SI, and NiBA-SI) and large intestine (MnBA-LI, CoBA-LI, and NiBA-LI); and total bioaccessibility (MnBA, CoBA, and NiBA) in vegetables and fruits (mean ± standard deviation, SD; %).

Vegetables / Fruits	Mn (mg/kg)	MnBA-SI (%)	MnBA-LI (%)[#****]	Total MnBA (%)[#*]
Vegetables				
Zucchin	2.20 ± 0.27	14.0 ± 6.42 [ABCDEFFHIJK]	8.16 ± 8.95[AB]	22.2 ± 11.6 [ABCDEFGHIJK]
Capsicum	1.88 ± 0.55	18.8 ± 5.88 [ALMNOPQ]	8.83 ± 10.3[CD]	27.6 ± 12.6 [AMNOKPRS]
Carrot	1.36 ± 0.45	19.6 ± 8.11 [BRSTUV]	13.3 ± 14.6[AEF]	32.9± 18.2 [BTUVWX]
Potatoe	2.10 ± 0.62	40.4 ± 10.2 [CLRWXYZA′B′C′D′]	5.52 ± 7.24 [CEGHIJKL]	45.9 ± 11.4 [CMYZA′B′C′D′E′F′]
Sweet potatoe	1.96 ± 0.33	19.2 ± 1.30 [DWE′F′G′H′I′J′K′]	9.49 ± 10.9[G]	28.7 ± 10.9 [DYG′H′I′J′K′L′]
Eggplant	2.64 ± 0.66	9.54± 5.30 [EMSXE′L′M′N′O′P′Q′]	8.94 ± 11.1[FM]	18.5 ± 12.5 [ENAG′M′N′O′P′Q′R′]
Onion	2.57 ± 0.62	17.0 ± 8.59 [EYF′L′R′S′T′U′V′]	13.4 ± 17.0[H]	30.4 ± 18.6 [FZA′M′S′T′U′]
Cauliflower	2.42 ± 0.76	22.7 ± 10.1 [EZG′M′R′W′X′Y′Z′]	13.1 ± 16.0[I]	35.8 ± 19.3 [GOB′H′N′V′W′X′]
Spinach	3.64 ± 1.88	46.7 ± 14.9 [ENTA′H′N′S′W′A′′B′′C′′]	10.9 ± 13.1[J]	57.6 ± 18.4 [HPC′I′O′S′V′Y′Z′]
Garlic	2.87± 0.50	41.7 ± 18.9 [EOUB′I′P′T′X′A′′D′′E′′]	10.3 ± 13.1[KNO]	52.0 ± 23.6 [IQD′J′P′T′W′A′′B′′]
Tomatoe	1.95 ± 0.81	25.4± 12.2 [EPC′J′P′U′Y′B′′D′′]	9.82 ± 13.2[NP]	35.2 ± 18.0 [JRE′K′Q′Y′A′′C′′]
Cabbage	1.40 ± 0.26	26.4 ± 6.20 [EQVD′K′Q′V′Z′C′′E′′]	16.9 ± 19.1 [BDLMOP]	43.4 ± 19.7 [KSL′R′U′X′Z′B′′C′′]
Fruits				
Apple	2.37 ± 0.51	5.57 ± 3.39[abcde]	10.5 ± 10.7[a]	16.1 ± 11.1[abcde]
Banana	2.03 ± 0.38	24.6 ± 6.22[afgh]	8.75 ± 9.80[b]	33.4 ± 11.1[afgh]
Orange	1.50 ± 0.21	16.8 ± 3.11[bfijk]	15.6 ± 17.8[bcd]	32.3 ± 17.0[bij]
Grapes	1.20 ± 0.19	25.9 ± 10.4[cilm]	14.2 ± 17.0[ef]	40.1 ± 19.7[cfikl]
Plum	1.89 ± 0.35	11.7 ± 4.29[dgjln]	8.23 ± 11.2[ace]	19.9 ± 12.1[dgjkm]
Peach	1.59 ± 0.18	19.3 ± 6.37[ehkmn]	9.70 ± 12.7[df]	29.0 ± 15.2[ehlm]
**Vegetables / Fruits**	**Co (µg/kg)**	**CoBA-SI (%)[#*]**	**CoBA-LI (%)[#*]**	**Total CoBA (%)[#*]**
Vegetables				
Zucchini	6.07 ± 1.81	30.7 ± 9.00[ABCDEFGH]	6.73 ± 5.06[ABCD]	37.4 ± 9.67[ABCDEFGHI]
Capsicum	7.05 ± 2.60	53.6 ± 20.1 [AIJKLMNOPQ]	7.10 ± 6.24[EFGHI]	60.7 ± 21.8[AJKLMNOPQR]
Carrot	5.55 ± 1.79	32.7 ± 15.9 [IRSTUVW]	9.48 ± 6.05[AEJKLMNOPQR]	42.2 ± 17.5[JSTUVWX]
Potatoe	5.91 ± 1.57	36.2 ± 7.13[BJXYZA′B′C′D′]	4.91 ± 4.74[FJSTU]	41.1 ± 8.64[BKYZA′B′C′D′]
Sweet potatoe	3.80 ± 0.42	27.9 ± 7.40 [KXE′F′G′H′I′J′]	14.5 ± 8.79[BGKSUVWXYZ]	42.4 ± 10.7[CLE′F′G′H′I′]
Eggplant	9.87 ± 3.14	28.9 ± 13.9 [LRK′L′M′N′O′P′]	7.53 ± 7.26[LUA′]	36.4 ± 15.8[MSJ′K′M′N′O′P′]
Onion	9.73 ± 3.75	20.6 ± 9.23 [CMSYE′K′Q′R′S′T′U′]	6.47 ± 5.23[MB′]	27.1 ± 11.0[DNTYE′J′Q′R′S′]
Cauliflower	13.6 ± 3.66	38.0 ± 16.6 [DNZF′L′Q′V′W′X′Y′]	4.81 ± 3.36[CHNWC′D′E′F′]	42.8 ± 17.2[EOZK′Q′T′U′V′W′]
Spinach	9.74 ± 6.64	23.5 ± 7.13 [EOTA′G′M′R′V′Z′A′′]	6.64 ± 4.77[OXC′G′]	30.1± 9.26[FPUA′F′L′T′X′Y′]
Garlic	8.84± 0.90	24.2 ± 8.51 [FPUB′H′N′S′W′B′′C′′]	6.16 ± 4.23[PYD′H′]	30.4 ± 9.86[GQVB′G′M′U′Z′A′′]
Tomatoe	6.75 ± 3.19	45.2± 14.8 [GQVC′I′POT′X′Z′B′′]	7.08 ± 5.20[QTZE′I′]	52.3 ± 16.0[HRWC′H′N′R′V′X′Z′B′′]
Cabbage	4.35 ± 1.58	51.2 ± 15.1 [HWD′J′P′U′Y′A′′C′′]	13.0 ± 7.77[DIRUA′B′F′G′H′I′]	64.2 ± 17.2[IXD′I′O′S′W′Y′A′′B′′]
Fruits				
Apple	6.85 ± 2.56	13.7 ± 7.72[abcde]	11.2 ± 8.20[ab]	24.9 ± 9.10[abcd]
Banana	4.50 ± 1.35	25.8 ± 6.47[afghi]	11.0 ± 6.73[cd]	36.8 ± 10.2[aefgh]

Orange	6.21 ± 2.14	22.9 ± 5.93^{bfjkl}	9.19 ± 6.35	32.1 ± 8.87^{beijk}
Grapes	6.39 ± 6.63	55.7 ± 22.5^{CGMN}	13.0 ± 11.8	68.7 ± 18.6^{cfihlm}
Plum	5.36 ± 1.81	14.7 ± 10.9^{dhkmo}	7.48 ± 5.24^{ac}	22.2 ± 11.2^{gjhln}
Peach	4.74 ± 1.09	6.17 ± 7.48^{eilno}	8.08 ± 5.79^{bd}	14.2 ± 9.05^{dhklmn}

**Vegetables / Fruits**	**Ni (μg/kg))**	**NiBA-SI (%)$^{\#*}$**	**NiBA-LI (%)$^{\#*}$**	**Total NiBA (%)$^{\#*}$**
Vegetables				
Zucchini	157.3 ± 61.6	23.9 ± 22.6^{ABCDEFGHIJK}	2.63 ± 2.59 ^{ABCDEFGHI}	26.6 ± 24.2 ^{ABCDEFGHIK}
Capsicum	46.3 ± 22.0	59.1 ± 12.7^{ALMNOPQR}	8.35 ± 7.60^{AJKLMN}	67.5± 15.6^{ALMNOPQ}
Carrot	69.4 ± 33.4	54.5 ± 24.0^{BSTUVW}	5.98 ± 5.77^{BJOPQRST}	60.4 ± 23.6^{BRSTUVW}
Potatoe	73.3 ± 37.9	47.0 ± 12.5$^{CLRXYZA'B'C'}$	3.87 ± 4.39$^{KOUVWXYZA'B'}$	50.9 ± 12.6$^{CLRXYZA'}$
Sweet potatoe	41.4 ± 3.87	43.0 ± 13.4$^{DMXD'E'F'G'}$	8.99 ± 6.59$^{CPUC'D'}$	52.0 ± 14.1$^{DMSB'C'D'E'}$
Eggplant	91.2 ± 28.2	26.8 ± 22.3$^{ENSYH'I'J'K'L'M'}$	10.1 ± 14.5$^{DVE'F'}$	36.8 ± 23.4$^{ENTXB'F'G'H'I'J'K'}$
Onion	45.2 ± 22.4	37.3 ± 19.8$^{FOTZH'N'O'P'Q'}$	10.6 ± 6.64$^{ELQWE'G'H'I'}$	47.9 ± 19.8$^{FOUF'L'M'N'O'}$
Cauliflower	57.4 ± 30.5	39.5 ± 27.5$^{GPUI'R'S'T'U'}$	10.6 ± 9.98$^{FRXJ'K'L'}$	50.1 ± 26.1$^{GPVG'Q'R'S'T'}$
Spinach	452 ± 459	22.4 ± 25.8$^{HQWA'D'J'N'R'V'W'X'}$	3.84 ± 5.76$^{MSYC'F'G'J'M'N'O'}$	26.2 ± 27.5$^{HQWTYC'H'L'Q'U'V'W'}$
Garlic	54.7 ± 15.3	57.4± 13.2$^{IRB'E'K'0'S'V'}$	7.98 ± 8.63$^{GZH'K'M'}$	65.4 ± 14.6$^{IUZD'I'M'R'U'}$
Tomatoe	50.5 ± 25.5	53.1 ± 29.8$^{JF'L'P'T'W'}$	6.22 ± 5.88$^{HNA'D'I'L'N'P'}$	59.4 ± 28.3$^{JJ'N'S'V'}$
Cabbage	32.5 ± 9.23	56.9 ± 11.8$^{KC'G'M'Q'U'X'}$	10.5 ± 9.55$^{ITB'O'P'}$	67.4 ± 12.6$^{KA'E'K'O'T'W'}$
Fruits				
Apple	221 ± 65.3	3.10 ± 1.82^{abcde}	2.38 ± 1.67^{abcd}	5.48 ± 2.32^{abcde}
Banana	46.4 ± 7.78	52.3 ± 17.1^{aefg}	8.02 ± 7.03^{aef}	60.4 ± 18.1^{afg}
Orange	42.7 ± 35.5	43.8 ± 15.4^{behij}	13.0 ± 9.51^{beghi}	56.8± 17.7^{bhij}
Grapes	36.0 ± 20.3	56.1 ± 17.6^{chkl}	8.57 ± 7.06^{cgj}	64.7 ± 17.5^{chkl}
Plum	19.8 ± 7.52	88.4 ± 7.37^{dfikm}	7.81 ± 7.72^{dhk}	96.2 ± 5.22^{dfikm}
Peach	46.1 ± 15.3	70.4 ± 15.4^{egjlm}	4.47 ± 6.89^{fijk}	74.9 ± 15.5^{egjlm}

# The presence of columns labelled with identical superscript capital letters for MnBA, CoBA and NiBA values in different vegetables indicate statistically significant differences on the Kruskal-Wallis and Mann-Whitney U test ($p < 0.05$).

* The presence of columns labelled with identical lowercase letters for MnBA, CoBA and NiBA values in different fruits indicate statistically significant differences on the Kruskal-Wallis and Mann-Whitney U test ($p < 0.05$).

Table S8. Mean and range of Mn (mg/kg) and Co and Ni (µg/kg) concentrations, corresponding bioaccessibility values (MnBA, CoBA, and NiBA, respectively; %) reported in foods, as well as daily dietary intakes and risk assessments established for these elements in other studies.

Food group	Mn, X ± SD# (mg/kg)	Mn, Range (mg/kg)	MnBA (%)	Main outcomes (MnBA)	Daily dietary intakes and risk assessments	Reference
Nuts (Bulgaria)	36.4	21.0 – 55.0	67.0 – 79.0 (72.8: gastric phase)<br>20.0 – 41.0 (29.8: intestinal phase)	The high solubility of Mn in the gastric phase (acidic pH) was considerably reduced at the neutral pH of the intestinal step.	-	[69]
Gluten free food (dried weight d.w.; Italy)	2.90	0.47-2.90	-	-	Daily intake per serving (serving size of 100 g: 0.20 mg, which supplies 13% and 16% of the recommended dietary intakes (RDI) for men and women, respectively); mean risk index value of 0.030.	[92]
Vegetables (fresh weight: f.w.; China)	5.49	1.02- 13.0	39.5 - 98.0 (small intestinal phase)<br>4.10 - 83.8 (colon phase)	An *in vitro* gastrointestinal method combined with the SHIME model was used to simulate the subsequent colon phase; in this colonic phase, the human gut microbiota reduced the dissolution of Mn and, consequently, its bioaccessibility (MnBA) in vegetables. Spinach was found to be the best source of bioaccessible Mn.	-	[60]

Rice varieties (Nigeria)	60.9 (un-parboiled rice)<br>10.2 (parboiled rice)	49.5-81.2<br><br>9.11-11.0	-	-	Mn concentrations were considerably reduced by the parboiling treatment of rice samples; consequently, potential health risks associated with rice consumption in relation to Mn exposure were diminished by this hydrothermal process.	[46]
Beef cuts (f.w.; Uruguay)	0.14	0.11-0.21	-	-	The estimated daily intake (EDI) of Mn (serving size: 100 g) is 00.14 mg, which corresponds to 0.77% and 0.61%f the recommended daily intakes for women and men, respectively; thus, beef cuts do not pose health risks regarding Mn exposure.	[42]
Rice varieties (d.w.; Spain)	9.00	2.45 – 13.6	3.45 – 26.8 (13.1; solubility)<br>1.07 – 22.8 (4.11; dializability)	Dialyzability assay; brown rice varieties exhibited a low MnBA, primarily due to the negative interaction exerted by rice fiber.	-	[65]
Cheese products (Greece)	0.23	0.03-0.54	-	-	The EDI per serving (22 g/day) of Mn is 0.06 mg, indicating no potential health	[94]

					risk.	
Fortified infant foods (Brazil)		<LQO-4.80	<LOQ-4.48	An *in vitro* digestion system was applied; as dietary fiber and phytic acid levels increased, the bioaccessible fractions of Mn decreased, whereas vitamin C enhanced Mn bioaccessibility.	-	[63]
Vegetables and fruits (d.w.; Turkey)	47.7	5.67-96.1	75.0–150 (gastric phase)<br>34.0–176 (gastro-intestinal phase)	An *in vitro* gastrointestinal digestion process was used; the bioaccessible fractions of Mn in both the gastric and gastrointestinal phases varied depending on the fruit and vegetable species.	-	[71]
Traditional meals of Mediterranean diet (Italy)	1.60	0.14-12.3	-	-	The EDI was 0.63 mg/day; the samples provide 6.9% and 8.8% of the recommended daily intakes for men and women, respectively. The health risk index (HRI) was lower than the RfD_{ing}.	[91]
Plant-based yogurts (Italy)	-	0.50-3.40	30	The INFOGEST protocol revealed a wide variation in MnBA.	One serving (170 g) provides 7.37% and 14.7% of the recommended daily intake for adults and children aged 4–8 years, respectively.	[67]

Nuts (f.w)	39.7 (raw samples)	-<br>7.07 ± 88.7	27.7 (raw samples)<br>19.7 (roasted samples)	The highest MnBA was observed in cashews 48.5% raw form; 33.3% in roasted form. Among the analyzed nuts and seeds (almond, hazelnut, chickpea, and cashew), the roasting process decreased the MnBA in all samples.		[41]
Rice (Malaysia)	-	-	-	When rice was subjected to a combined washing, parboiling, and absorption treatment, the native Mn content was reduced by 67.8%.	For washed rice, the hazard quotient (HQ) for Mn was 0.41 for children (serving size: 70 g/day) and 1.01 for adults (serving size: 307 g/day); supplementary rice parboiling and absorption processes diminished the HQ values to 0.23 and 0.5, respectively.	[72]
Plant-based burgers (d.w.)<br>Meat-based burgers (d.w.; Brazil)	-	<LOQ – 22.6<br><LOQ	<LOQ-61.7	The INFOGEST 2.0 protocol showed wide variability in MnBA values among samples. The MnBA was greater than the limit of quantification (>LOQ) only for chickpea- and soybean-based burgers.	-	[61]
Lentils (United Kingdom)	11.1	10.9-11.3	91.3 (raw conventional lentils)<br>89.9 (raw organic lentils)	The simplified bioaccessibility extraction test (SBET) was used to evaluate Mn bioaccessibility. Cooking retained more than 50% of Mn	One serving size (80 g) contributes to 30.7% of the recommended dietary intakes	[73]

				bioaccessibility, with no significant differences observed between organic and conventional lentils.		
Meats (Brazil)	295	215-415	-	-	Among the evaluated cooking methods (baking/muffining, roasting, and air frying), the latter emerged as the most effective in reducing exposure to potentially toxic elements.	[95]
Cocoa beans (d.w.; Ghana)	-	18.9-23.7	69.1 ± 2.09	An *in vitro* bioaccessibility assay was used, revealing a high percentage of Mn bioaccessibility.	The target hazard quotient (THQ) value for Mn was less than 0.1 (THQ < 0.1), indicating no potential health risk for either children or adults.	[23]
Vegetables (d.w.)	-	<4.87-17.7	-	-	The EDI exceeded the tolerable upper intake level (UL), indicating significant dietary risks.	[15]
Fruits (d.w.)	-	<1.87-14.2	-	-		
Cereals (d.w.; Poland)	-	<4.87-170	-	-		
Animal liver (f.w.; Turkey)	7.86	6.07-9.46; Mineral retention after cooking by pan-frying, boiling, *sous-vide*, and deep-frying (7.08)	150-257 (depending on the cooking method applied)	An *in vitro* oral-gastrointestinal digestion protocol was used; Mn retention and its release into the soluble fraction varied depending on both the cooking treatment and the type of liver, with sous-vide being the most effective	-	[31]

cooking method.

Food group	Co, X ± SD# (μg/kg)	Co, Range (μg/kg)	CoBA (%)	Main outcomes (CoBA)	Daily dietary intakes and risk assessments	Reference
Gluten free food (d.w.; Italy)	84.0	0.30-540	-	-	-	[92]
Rice (Malaysia)	-	-	-	An *in vitro* oral-gastrointestinal digestion method simulating the small intestine phase was applied; the bioaccessible fraction of Co ranged from 15 to 50 μg/kg (with a mean bioaccessible Ni fraction of 30.8 μg/kg).	No potential carcinogenic or non-carcinogenic health risks were identified for either adults or children.	[93]
Traditional meals of Mediterranean diet (Italy)	29.0	2.1-500	-	-	The mean estimated daily intake (EDI) was 2.4 μg/day, with the HRI remaining below the RfDing.	[91]
Plant- and animal-based yogurts (Italy)	-	<LOQ – 41.1	n.d -89.0<br>7.0-90.0 (plant-based yogurts)	The INFOGEST protocol revealed a wide variation in CoBA; notably, animal-based yogurt did not present quantifiable bioaccessible levels.	-	[67]
Rice (Malaysia)	-	-	-	When rice was subjected to combined washing, parboiling, and absorption, the native Co content was reduced by 81.6%.	-	[72]
Plant-based meat (f.w.; Brazil)	34.8	3.6-109	33-80 (plant-based samples);<br><LOQ-66.6 (meat samples)	The INFOGEST 2.0 protocol.	No health concerns were identified for adults (highest exposure [HE]: 0.2 μg/kg-	[24]

					day); however, further research is required for children (HE: 0.8 μg/kg-day).	
Vegetables (d.w.)<br>Fruits (d.w.)<br>Cereals (d.w.; Poland)	-<br><br>--	<LOQ-<195<br><20.0-<195<br><20.0-<195	-<br><br>--	-<br><br>--	The EDI exceeded the typical range (5-8 μg/day), indicating potential non-carcinogenic risks under the assumption of high bioaccessibility (BA).	[15]

**Food group**	**Ni, X ± SD#** **(μg/kg)**	**Ni, Range** **(μg/kg)**	**NiBA (%)**	**Main outcomes (NiBA)**	**Daily dietary intakes and risk assessments**	**Reference**
Foodstuffs (Slovenia)<br>Cacao drink	2,890<br>5,200	200-5,950<br>5,000-5,400	-	-	The consumption of 200 g of soy yogurt or tofu contributes 1,200 or 400 μg of Ni, respectively; additionally, cooking in stainless steel pots results in Ni leaching.	[16]
Gluten free food (d.w.; Italy)	4,400	16.0-14,000	-	-	The daily intake per serving (serving size: 100 g was 440 μg, supplying 63% of the daily reference intake; the mean risk index value was 0.316.	[92]
Cheese products (Greece)	220	70.6-657	-	-	The EDI per serving (22 g/day) of Ni is 0.48 μg, indicating no potential health hazard.	[94]

Animal-based foods (f.w.; undefined country)	-	13.0-4,150	<LOQ-83% (different foods)	Future studies are required to improve our understanding of the factors governing Ni bioaccessibility, particularly in foods rich in this element (e.g., legumes, chocolate, nuts, and soy by-products).	The EDI ranged from 7 to 172 μg/day, with chronic dietary exposure being noted.	[66]
Plant-based foods (f.w.; undefined country)		24.0-44,100				
Beef cuts (f.w.; Uruguay)	56.0	124-198	-	-	The EDI of Ni (serving size: 100 g is 5.6 μg; beef cuts do not pose health risks even if more than one serving is consumed per day.	[42]
Rice (d.w.; Spain)	168 (raw form)	63.0 – 405	-	*In vitro* gastrointestinal digestion simulation was used; bioaccessible Ni concentrations in rice varieties cooked in stainless steel cookware were similar to those observed in samples cooked in glass containers.	Although cooking rice in stainless steel cookware can increase the total Ni content, its bioaccessibility remains low; therefore, it does not represent a hazard to human health.	[45]
	160 (cooked in glass container	67.0-425	82.5 (rice cooked in glass container)			
	215 (cooked in stainless steel container)	90.0-421	78% (rice cooked in stainless steel container)			
Traditional meals of Mediterranean diet (Italy)	200	18.0-1,500	-	-	The mean estimated daily intake (EDI) was 58.0 μg/day; a single serving (100 g) provides 58% of the recommended daily intake, with the HRI remaining	[91]

					below the RfDing.	
Rice (Iran)	480-± 360	10.0-1,140	-	-	The EDI was 3.4 x 10^{-5} mg/kg-day; a lifetime carcinogenic risk for potentially toxic elements (including Ni values of 2.99 x 10^{-3} and 7.47 x 10^{-4} for children and adults, respectively) was observed. Furthermore, children exhibited a higher non-carcinogenic risk than adults.	[22]
Plant-based beverages (Italy)	-	-	51-107	The INFOGEST 2.0 protocol.	-	[70]
Food products (undefined country)	-	<10.0-1,710	-	-	The consumption of cocoa, chocolate, highly processed foods, and canned products should be reduced due to their elevated Ni levels.	[21]
Plant-based meat (f.w.; Brazil)	304.4	20-1,096	34-77 (plant-based samples); <LOQ-43.6	The INFOGEST 2.0 protocol.	No health concerns were identified for adults (Highest exposure [HE]: 1.9 μg/kg-day); however, further research is required for children, who exhibited a higher exposure (HE: 7.7 μg/kg-day}.	[24]

Plant-and animal-based yogurts (Italy)	-	<LOQ – 491	7.00 -89.9 (plant-based yogurts)	The INFOGEST protocol revealed a wide variation in NiBA; animal-based yogurt did not present quantifiable bioaccessible levels.	-	[67]
Rice (Malaysia)	-	-	-	When rice was subjected to combined washing, parboiling, and absorption, the native Ni content was reduced by 85.4%.	For washed rice, the hazard quotient (HQ) for Ni was 1.1 for children (serving size: 70 g/day) and 2.7 for adults (serving size: 307\ g/day); subsequent rice parboiling and absorption processes diminished these HQ values to 0.18 and 0.45, respectively.	[72]
Cocoa beans (d.w.; Ghana)	-	10.2-11.8	70.5 ± 2.01	An *in vitro* bioaccessibility assay was used, revealing a high percentage of Ni bioaccessibility.	The target hazard quotient (THQ) for Ni was below 0.1 (THQ < 0.1), indicating no potential health risks for either children or adults.	[25]

[#]Standard deviation.

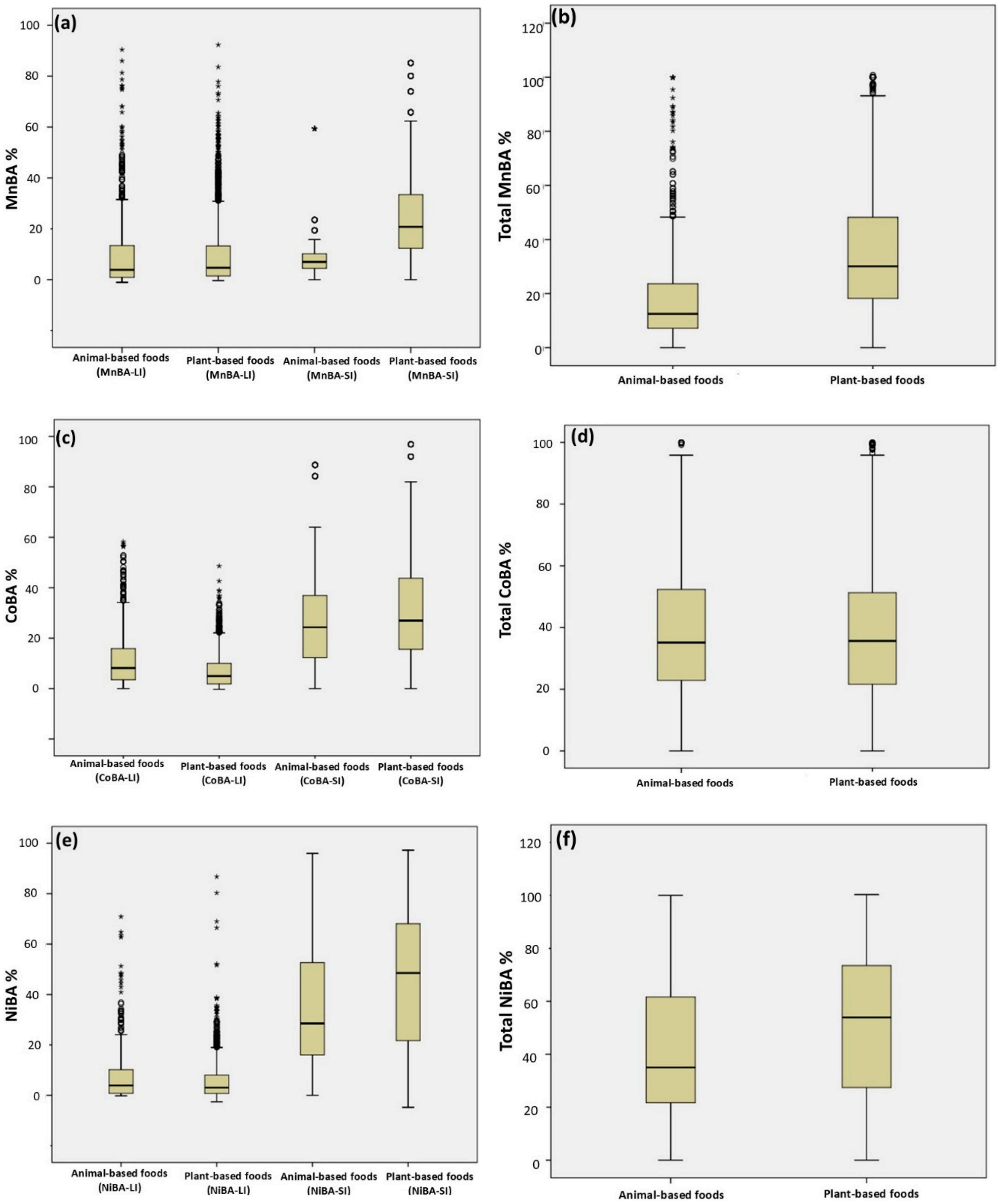

Figure S1. Box-and-Whisker plots for total and partial bioaccessibility values (%; total BA; BA in the small intestine, and in the large intestine: BA-SI, and BA-LI; respectively) of the trace metals analyzed in plant- and animal-based foods: (**S1a**) Mn bioaccessibility in the small, and in the large intestine (MnBA-SI, and MnBA-LI; respectively); (**S1b**) Total Mn bioaccessibility (Total MnBA); (**S1c**) CoBA-SI, and CoBA-LI; (**S1d**) Total CoBA; (**S1e**) NiBA-SI, and NiBA-LI; (**S1f**) Total NiBA.

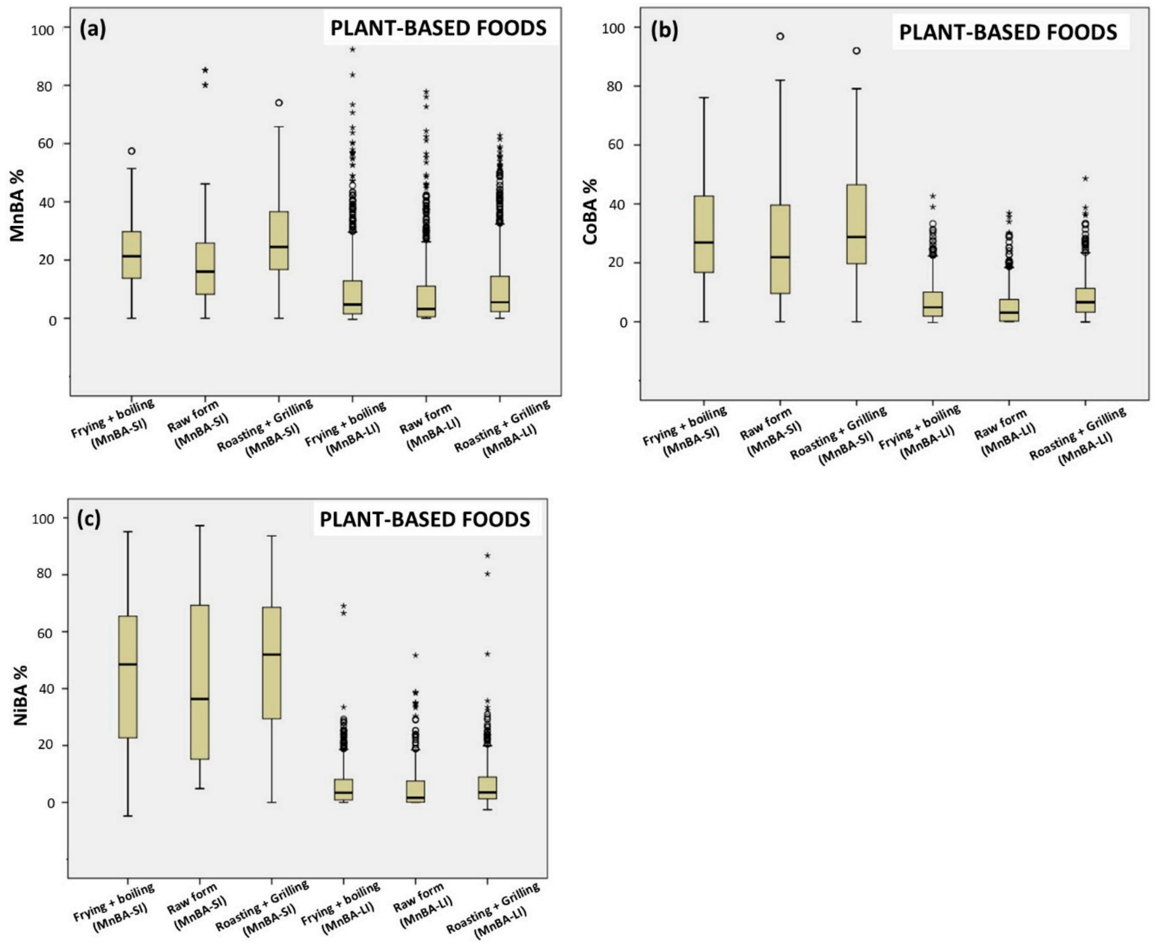

Figure S2. Box-and-Whisker plots for partial bioaccessibility values (BA in the small intestine, and in the large intestine: BA-SI, and BA-LI; respectively) of the trace metals analyzed in plant-based foods according to grouped cooking techniques (frying + boiling, raw form, and roasting + grilling): (**S2a**) Mn bioaccessibility in the small, and in the large intestine (MnBA-SI and MnBA-LI, respectively); (**S2b**) CoBA-SI and CoBA-LI; (**S2c**) NiBA-SI and NiBA-LI.

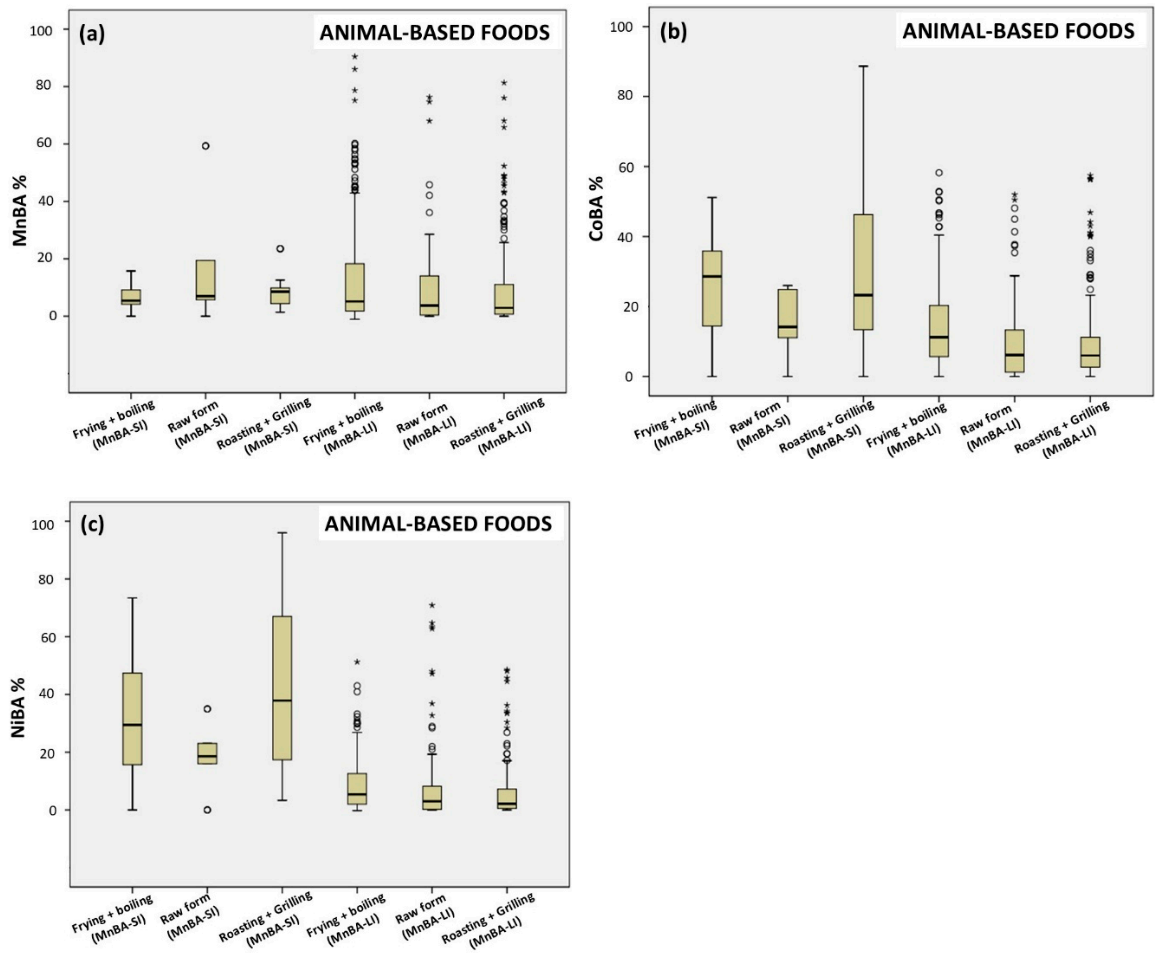

Figure S3. Box-and-Whisker plots for partial bioaccessibility values (BA in the small intestine, and in the large intestine: BA-SI, and BA-LI; respectively) of the trace metals analyzed in animal-based foods according to grouped cooking techniques (frying + boiling, raw form, and roasting + grilling): (**S3a**) Mn bioaccessibility in the small, and in the large intestine (MnBA-SI and MnBA-LI, respectively); (**S3b**) CoBA-SI and CoBA-LI; (**S3c**) NiBA-SI and NiBA-LI.

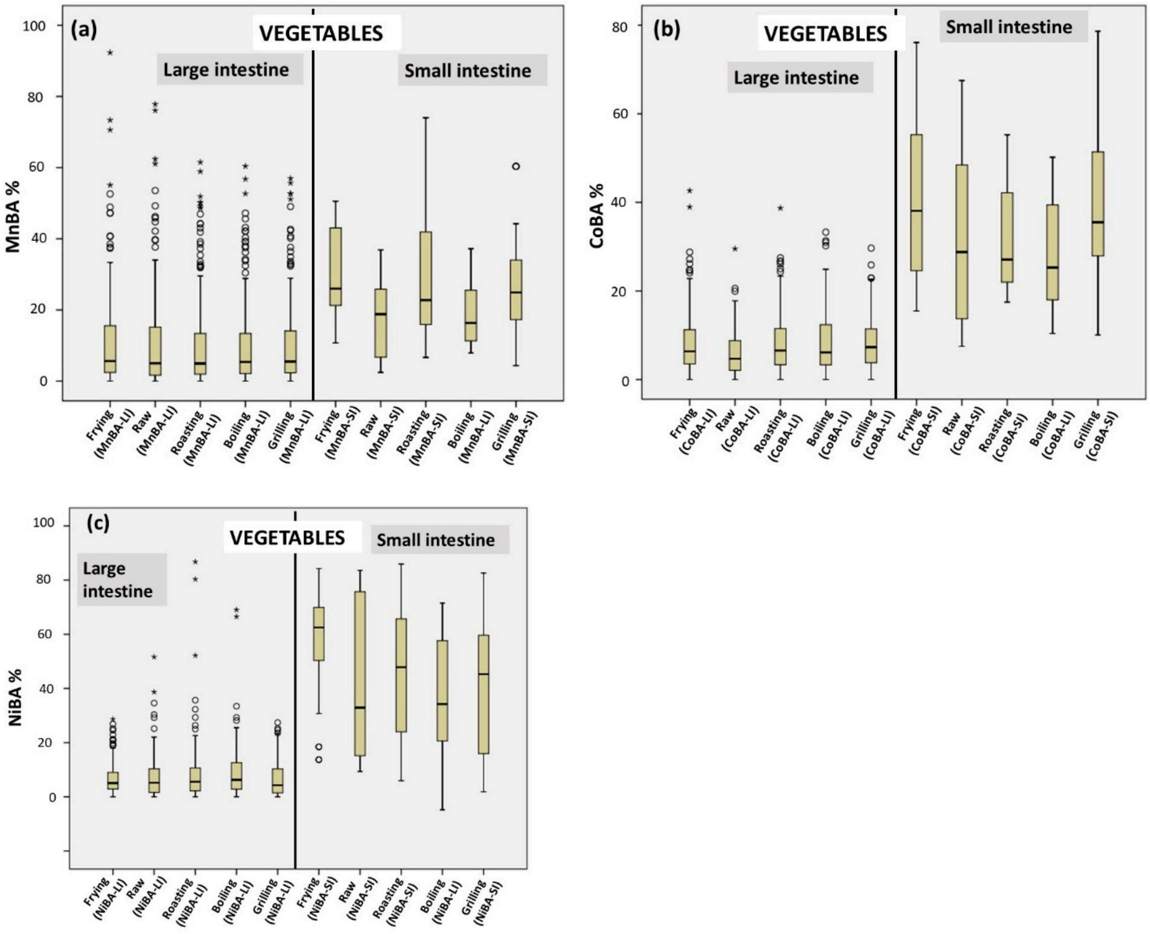

Figure S4. Box-and-Whisker plots for partial bioaccessibility values (BA in the small intestine, and in the large intestine: BA-SI, and BA-LI; respectively) of the trace metals analyzed in vegetables according to cooking technique: (**S4a**) Mn bioaccessibility in the small, and in the large intestine (MnBA-SI and MnBA-LI, respectively); (**S4b**) CoBA-SI and CoBA-LI; (**S4c**) NiBA-SI and NiBA-LI.

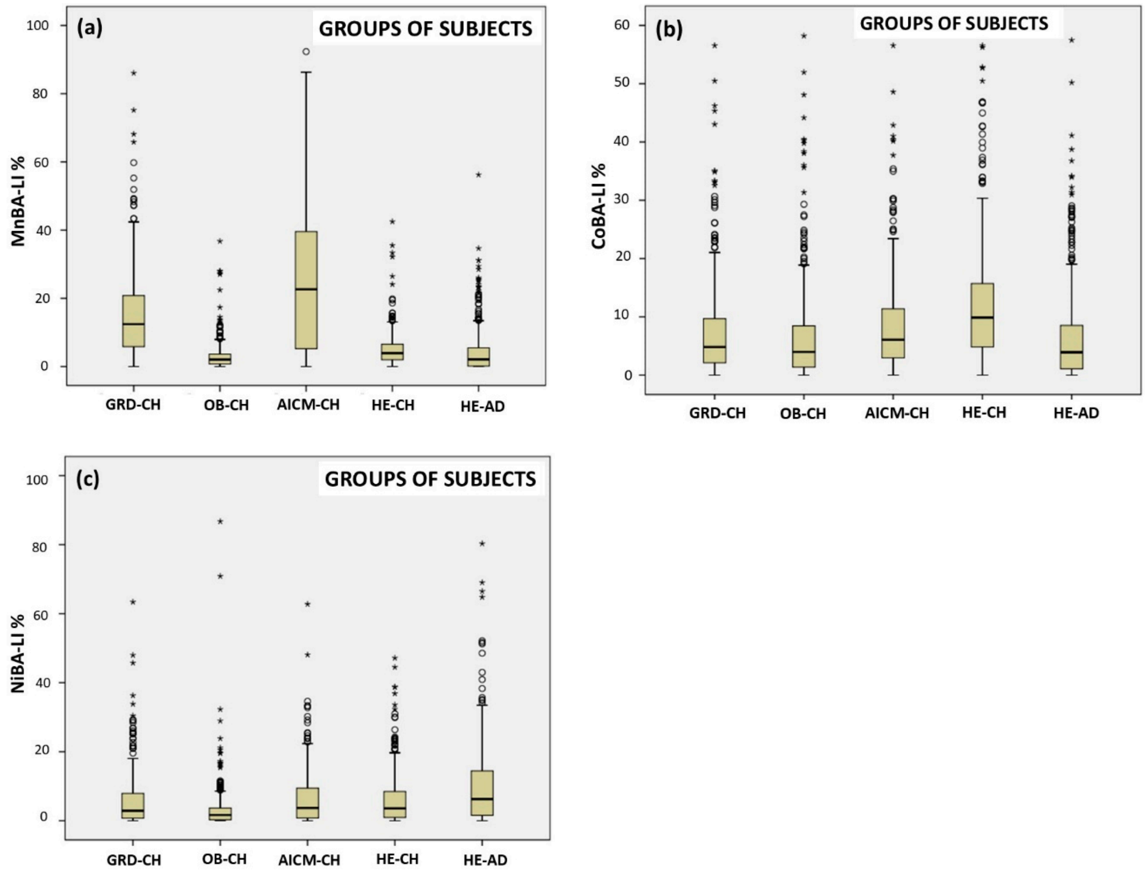

Figure S5. Box-and-Whisker plots for bioaccessibility values in the large intestine (BA-LI) of the trace metals analyzed in all foodstuffs using fecal inoculum from healthy adults (HE-AD), healthy children (HE-CH), children with gluten-related disorders (GRD-CH), children with obesity (OB-CH), and children with allergy/intolerance to cow's milk proteins (AICM-CH): (**S5a**) Mn bioaccessibility in the small, and in the large intestine (MnBA-SI and MnBA-LI, respectively); (**S5b**) CoBA-SI and CoBA-LI; (**S5c**) NiBA-SI and NiBA-LI.
